# FMOe: Preprocessing and Visualizing Package of the Fragment Molecular Orbital Method for Molecular Operating Environment and Its Applications in Covalent Ligand and Metalloprotein Analyses

**DOI:** 10.1021/acs.jcim.4c01169

**Published:** 2024-09-05

**Authors:** Hirotomo Moriwaki, Yusuke Kawashima, Chiduru Watanabe, Kikuko Kamisaka, Yoshio Okiyama, Kaori Fukuzawa, Teruki Honma

**Affiliations:** † Center for Biosystems Dynamics Research, RIKEN, 1-7-22 Suehiro-cho, Tsurumi-ku, Yokohama, Kanagawa 230-0045, Japan; ‡ Department of Physical Chemistry, School of Pharmacy and Pharmaceutical Sciences, Hoshi University, 2-4-41 Ebara, Shinagawa-ku, Tokyo 142-8501, Japan; § JST PRESTO, 4-1-8, Honcho, Kawaguchi, Saitama 332-0012, Japan; ∥ Department of Computational Science, Graduate School of System Informatics, Kobe University, 1-1 Rokkodai, Nada-ku, Kobe, Hyogo 657-8501, Japan; ⊥ Graduate School of Pharmaceutical Sciences, Osaka University, 1-6 Yamadaoka, Suita, Osaka 565-0871, Japan

## Abstract

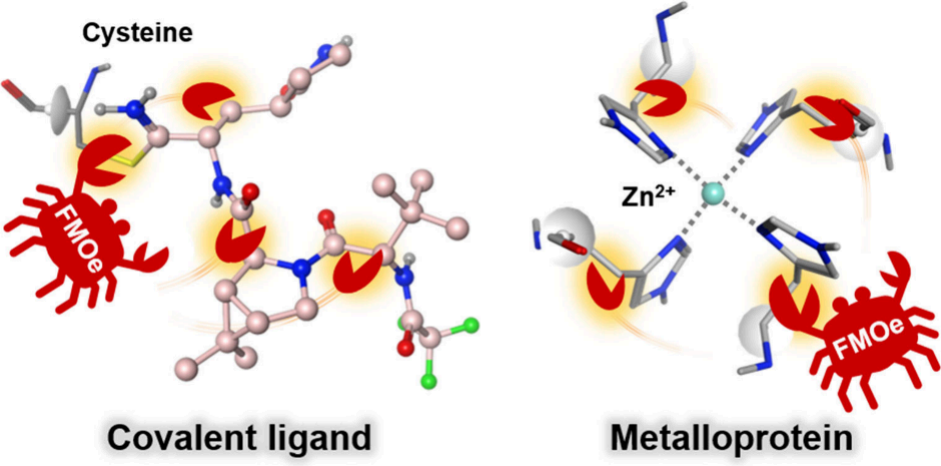

The fragment molecular orbital (FMO) method is an efficient quantum chemical calculation technique for large biomolecules, dividing each into smaller fragments and providing interfragment interaction energies (IFIEs) that support our understanding of molecular recognition. The *ab initio* fragment MO method (ABINIT-MP), an FMO processing program, can automatically divide typical proteins and nucleic acids. In contrast, small molecules such as ligands and heterosystems must be manually divided. Thus, we developed a graphical user interface to easily handle such manual fragmentation as a library for the Molecular Operating Environment (MOE) that preprocesses and visualizes FMO calculations. We demonstrated fragmentation with IFIE analyses for the two following cases: (1) covalent cysteine–ligand bonding inside the SARS-CoV-2 main protease (M^pro^) and nirmatrelvir (Paxlovid) complex and (2) the metal coordination inside a zinc-bound cyclic peptide. IFIE analysis successfully identified the key amino acid residues for the molecular recognition of nirmatrelvir with M^pro^ and the details of their interactions (e.g., hydrogen bonds and CH/π interactions) via ligand fragmentation of functional group units. In metalloproteins, we found an efficient and accurate scheme for the fragmentation of Zn^2+^ ions with four histidines coordinated to the ion. FMOe simplifies manual fragmentation, allowing users to experiment with various fragmentation patterns and perform in-depth IFIE analysis with high accuracy. In the future, our findings will provide valuable insight into complicated cases, such as ligand fragmentation in modality drug discovery, especially for medium-sized molecules and metalloprotein fragmentation around metals.

## 1 Introduction

Since quantum chemical calculations of biological macromolecules are expensive, various approximate methods^1−3^ have been proposed aiming at linear scaling, such as the fragmentation approaches, which reduce the problem size by dividing a whole system into smaller units. The fragment molecular orbital (FMO) method proposed by Kitaura et al.^3^ is one of the most popular methods that have advances in straightforward fragmentation using projection operators without artificial caps,^1,2^ fragment polarization embedded in the environmental field from self-consistent fragment densities, and superior parallelization. Furthermore, interfragment interaction energy (IFIE) can be quantitatively estimated together with its energy components by pair interaction energy decomposition analysis (PIEDA).^4−6^ These analyses are increasingly used as a support tool for elucidating molecular recognition mechanisms in drug discovery^6−25^ and structural biology^26−31^ over the years. As seen in Table S1, FMO-processing programs^32−40^ themselves or their preprocessing graphical user interfaces (GUIs)^41−52^ can automatically divide typical biopolymers, such as proteins or nucleic acids, into small fragments according to predefined rules on natural amino acid residues or nucleotide units. On the other hand, for practical purposes, one will need to perform atypical fragmentation manually in the following cases. For example, to elucidate the binding mode between a ligand and a protein of interest in drug discovery, fragmentation of functional groups is also required for interaction analysis.^8,9,11−13,20,25,53^ In addition, the coordination metal and its surrounding amino acid residues, which play an essential role in protein conformation in structural biology, must be treated as a single large fragment to solve the appropriate electronic state.^54−65^ While previous studies have attempted to address the fragmentation patterns of various ligands and metalloproteins, the existing manual fragmentation functions are far from user-friendly. The process of generating input files, such as assigning each atom to a fragment and setting bond detached atoms (BDAs) and bond attached atoms (BAAs) for fragmentation points based on the concept of projection operators, is a complex task. This complexity hinders the study using manual fragmentation for users other than GUI developers. Therefore, we developed a library called “FMOe”^48^ using Molecular Operating Environment (MOE)^66^ to provide a simple interface for creating input files and visualizing the analysis results for FMO calculations using the *ab initio* fragment MO method program (ABINIT-MP).^36−38^ The unique benefits of FMOe are best demonstrated by its ease of use in manual fragmentation and fragment merging functions for ligands and around coordination metals. This tool simplifies a complex process of manual fragmentation and makes FMO calculations more accessible and efficient for drug discovery and structural biology researchers.

Recently, there has been a demand for functional group unit interaction analyses via the fragmentation of ligands from the perspective of structure-based drug design in the pharmaceutical industry. Using FMOe will meet these demands and simplify the handling of ligands with covalent bonds and medium-molecular-weight drug candidate compounds (e.g., large compounds and cyclic peptides) for the “diversification of modality”. In addition, functionally important transition metal ions must be explicitly treated because they play an essential role in tertiary structure formation and catalysis in metal-containing proteins such as Zn fingers and enzymes. In the past, FMO calculations have often been performed with metals removed,^28,62,67^ prioritizing the convergency and ease of IFIE analysis. However, when discussing the results of FMO calculations with structural biologists, it has been requested that FMO calculations be performed using the net experimental structure, which still incorporates metals.^54−65^ When dealing with large fragments, including middle-molecule ligands and coordination metals, the issues are that the computational cost is high and the resolution of interaction energy analysis is reduced due to the large fragment size. Thus, it is necessary to consider appropriate fragmentation methods that ensure both ease of interaction energy analysis and reduction of computational cost while maintaining the computational accuracy of the analysis. In this study, as an example of analysis using FMOe, we will execute FMO calculations in the following molecular systems: (1) a complex between the SARS-CoV-2 main protease (M^pro^) and a tripeptide-like inhibitor, nirmatrelvir (brand name: Paxlovid, code name: PF-07321332)^68,69^ covalently bonded with Cys145 of M^pro^; and (2) a cyclic peptide with a Zn^2+^ ion as the structural center.^70^

## 2 Implementation

**Figure 1:**
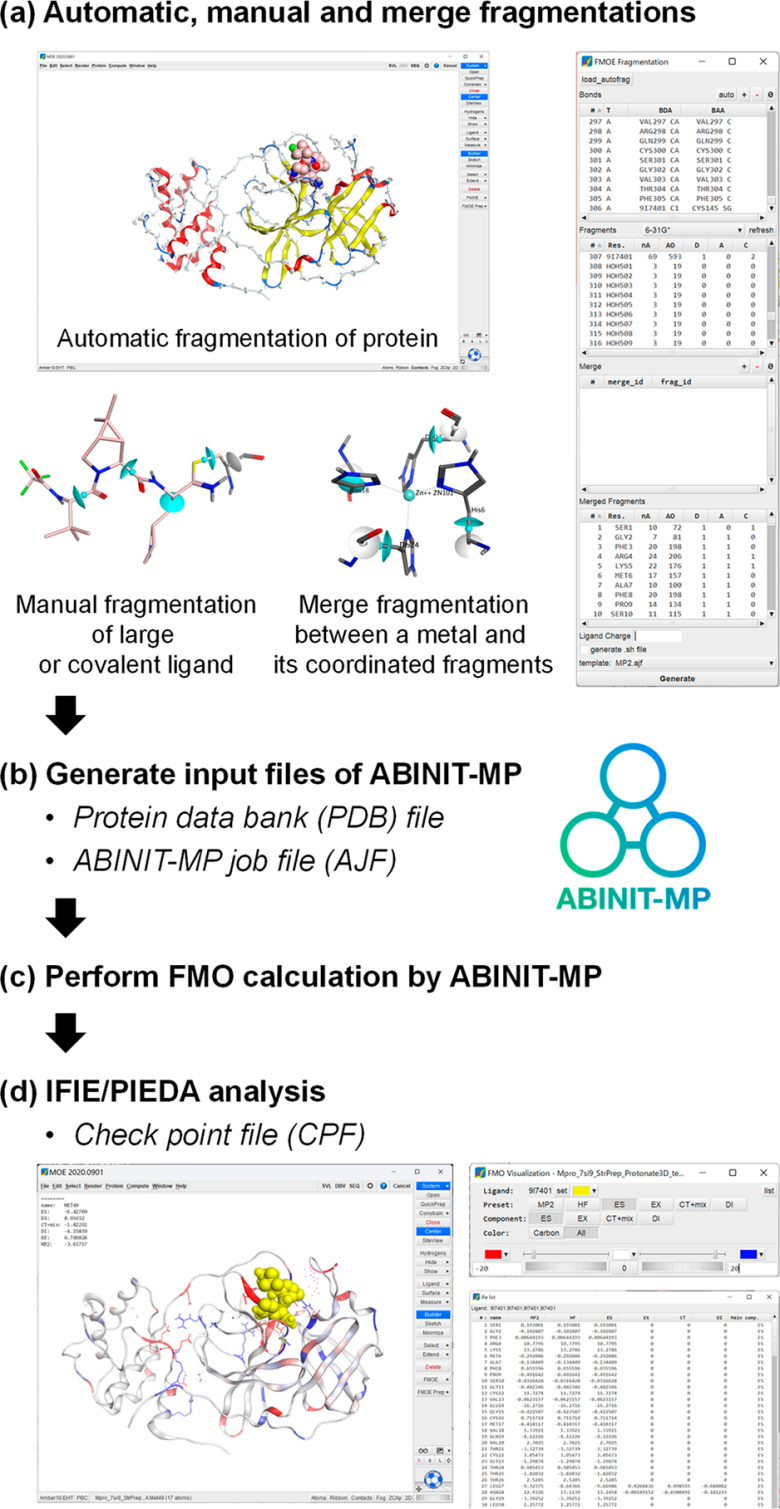
Framework of pre-/postprocessing for FMO calculation by FMOe.

MOE^66^ is a molecular modeling software program written in scientific vector language (SVL), a programming language for dealing with molecules, and the built-in SVL allows users to extend the software with more advanced functionality. Thus, FMOe was developed using MOE SVL and implemented for a preprocessing/calculation result visualization function for the FMO calculation using the ABINIT-MP program (Figure 1). As preprocession, FMOe provides several fragmentation functions of biological macromolecules and generates input files for FMO calculation. In particular, it supports automatic fragmentation for proteins containing non-natural amino acid residues and cyclic peptides and manual fragmentation. One can also extend the fragmentation rules with the SVL program. After fragmentation, one can easily modify multiple fragments of amino acid residues or water molecules that coordinate to a metal or ion to treat them as a single fragment. Postprocessing analysis of FMO calculation results includes IFIE/PIEDA analysis, which also easily allows for the summation of interaction energies of multiple fragments and the addition of any PIEDA energy components selected by the user. Detail descriptions of the FMO method and the functions and operating procedures of FMOe are presented in Section S1 of the Supporting Information.

## 3 Results and Discussion

### 3.1 Detailed Interaction Analysis of Functional Units of Covalent Ligand Using Manual Fragmentation Function

**Figure 2:**
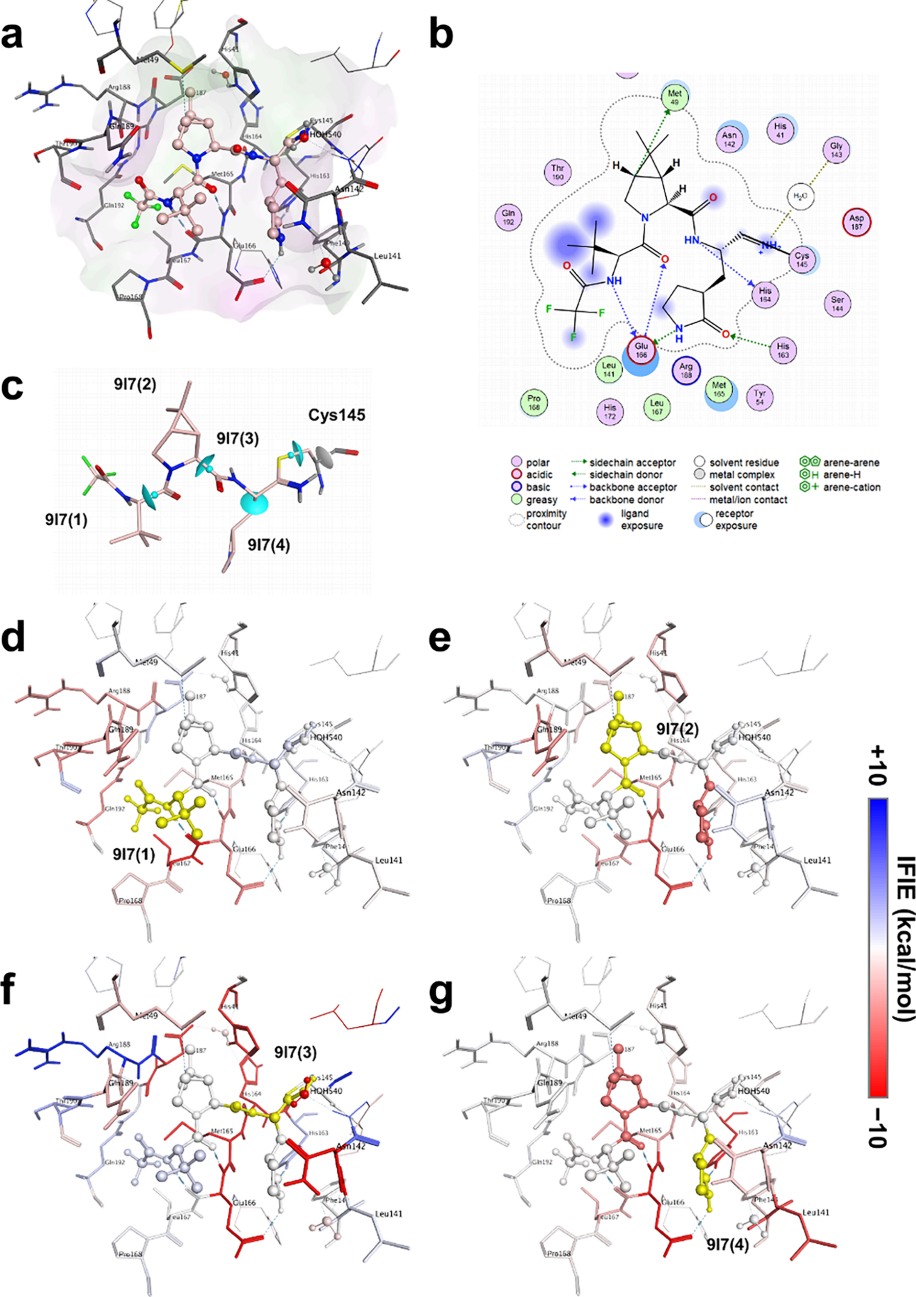
Interaction analysis between SARS-CoV-2 M^pro^ and nirmatrelvir. The ligand-binding pocket of nirmatrelvir of SARS-CoV-2 M^pro^ is represented by the gray stick model for amino acid residues and the pink ball and stick model for nirmatrelvir (a). Coordinate-based ligand interaction (2D) diagram (b). Fragment points of nirmatrelvir and Cys145 (c). The light blue disk indicates the bond detached atom (BDA)–bond attached atom (BAA), and the atoms on the side with the small light blue studs are BAAs (c). IFIE analysis of the ligand fragments (9I7(1)–9I7(4)) and the amino acid residues of SARS-CoV-2 M^pro^ (d)–(g).

As an example of a relatively sizable covalent inhibitor, FMO calculations were performed for the complex between SARS-CoV-2 M^pro^ and nirmatrelvir (PDB ID: 7SI9).^68,69^ Nirmatrelvir is an orally active SARS-CoV-2 M^pro^ inhibitor developed by Pfizer (Pty) Ltd. (USA). This covalent inhibitor binds directly to the enzyme catalyst Cys145 (Figures 2a and 2b). After the FMO calculation, IFIE/PIEDA analysis was performed to clarify the critical interaction between nirmatrelvir and each amino acid residue of M^pro^. First, the complex structure was pretreated via the following MOE procedure: the structure preparation function was applied to structurally complement the missing heavy atoms on the amino acid residues; consequently, hydrogen atoms on the complex were added using the Protonate3D function. Subsequently, structural optimization was performed with the Amber10:EHT force field using the energy minimization function of MOE. The atom constraints during structural optimization were as follows: all heavy atoms registered in the PDB were fixed; the complemented heavy atoms on amino acid residues and all hydrogen atoms were unconstrained. After structural optimization, M^pro^ and nirmatrelvir (3-letter code of ligand name in PDB: 9I7) were divided into each amino acid residue and four fragments (9I7(1)–9I7(4) in Figure 2c), respectively, according to the manual fragmentation procedure of FMOe (described in Section S1.2.1). The covalent bond site of Cys145 between M^pro^ and nirmatrelvir was fragmented into the main and side chains of cysteine. The FMO-MP2/6-31G* calculation was performed based on the input file obtained using FMOe. This FMO calculation result was registered in FMODB (https://drugdesign.riken.jp/FMODB/)^71,72^ with the code (FMODB ID) 4LQRN.

According to the IFIE/PIEDA analysis procedure of FMOe (described in Section S1.2.3), Figures 2d–2g, S5a, S5c, S5e, and S5g show the IFIE analyses between each amino acid residue of SARS-CoV-2 M^pro^ and nirmatrelvir; Figures S4a–S4p, S5b, S5d, S5f, and S5h show their PIEDA results, where the interaction energies with four ligand fragments of nirmatrelvir are represented (Figure 2c). For every ligand fragment (9I7(1)–9I7(4)), the tendency of the interaction energies with neighboring amino acid residues was different.

Figures 2d and S5a of the IFIE analysis show that the 9I7(1) fragment has the strongest attractive interaction with the Leu167 fragment (−22.1 kcal/mol). The other major attractive interacting fragments were Gln192 (−4.9 kcal/mol), Glu166 (−4.7 kcal/mol), Thr190 (−4.3 kcal/mol), Arg188 (−4.1 kcal/mol), and Gln189 (−3.8 kcal/mol). The reason for the strong interaction between the 9I7(1) and Leu167 fragment pair was suspected to be the NH–O hydrogen bond formed by the oxygen atom in the carbonyl group on the main chain of Glu166 (Leu167 fragment) and the hydrogen atom attached to a nitrogen atom in the amide group of 9I7(1). When verifying the PIEDA results (Figures S4a, S4i, and S5b), the ES component between the 9I7(1) fragment and the Leu167 fragment had a strong attractive interaction (−22.4 kcal/mol). In addition, the CT+mix component of its fragment pair, characteristic of hydrogen bond formation, exhibited a strong attractive interaction (−7.0 kcal/mol). In Gln189, the hydrogen atoms on the CA and CG carbon atoms in the main and side chains made CH–O hydrogen bonds with the oxygen atom of the amide of 9I7(1); a CH/π interaction was formed between the hydrogen atom on the trimethyl group of 9I7(1) and a π-orbital on the amide in the side chain of Gln189. Therefore, the CT+mix and DI components had weak attractive interactions of −2.0 and −3.9 kcal/mol, respectively (Figures S4i, S4m, and S5b). Here, 9I7(1) contains hydrophobic functional groups, such as trimethyl and trifluoromethane. Hence, the interaction between some amino acid residues in the vicinity and the DI component was confirmed (Figure S4m and S5b).

From Figures 2e and S5c of the IFIE analysis, the 9I7(2) fragment was attracted to Glu166 (−7.0 kcal/mol), Glu189 (−4.1 kcal/mol), Met165 (−3.5 kcal/mol), Leu167 (−3.2 kcal/mol), and His41 (−3.0 kcal/mol). Therefore, it can be confirmed that the carbonyl oxygen atom of 9I7(2) and the hydrogen atom attached to a nitrogen atom in the main chain of Glu166 were hydrogen-bonded based on the ES and CT+mix components (−7.5 and −1.6 kcal/mol, respectively; Figures S4b, S4j, and S5d). The 9I7(2) fragment contained hydrophobic functional groups like methane. Hence, the interaction between some amino acid residues in the vicinity (e.g., His41 and Met165) and the DI component can be confirmed (Figures S4n and S5d). Furthermore, it was confirmed from the analysis of the DI component that a CH/π interaction was formed between the hydrogen atom on the methyl group of 9I7(2) and a π-orbital on the indole ring of His41 (DI component: −3.8 kcal/mol), as a characteristic interaction.

From Figures 2f and S5e of the IFIE analysis, the 9I7(3) fragment was attracted to Asp187 (−32.4 kcal/mol), Glu166 (−25.8 kcal/mol), Met165 (−23.2 kcal/mol (including an oxygen atom in the main chain of His164)), HOH540 (−21.2 kcal/mol), His164 (−14.7 kcal/mol), Asn142 (−12.4 kcal/mol), Leu27 (−9.1 kcal/mol), and His41 (−8.8 kcal/mol). The ES component of PIEDA (Figures S4c and S5f) shows the presence of strong electrostatic interactions of the 9I7(3) fragment containing a charged imine with charged amino acid residues, polar amino acid residues near 9I7(3), and water (HOH540). However, when the CT+mix component of PIEDA was confirmed (Figures S4k and S5f), only the Met165 fragment and water (HOH540) exhibited attractive interactions. This result indicates that the CT+mix component can ensure the presence of hydrogen-bond-forming residues; that is, the hydrogen atom attached to a nitrogen atom of the amide group of 9I7(3) and the oxygen atom in the main chain of His164 (Met165 fragment) were NH–O hydrogen-bonded. Therefore, the Met165 fragment exhibited attractive interactions. In addition, analysis of the CT+mix components revealed that the NH of the 9I7(3) charged imine and the oxygen atom of the water molecule (HOH540) were NH–O hydrogen-bonded.

Figures 2g and S5g of the IFIE analysis show that the 9I7(4) fragment was attracted to His163 (−16.4 kcal/mol), Glu166 (−13.4 kcal/mol), Leu141 (−7.0 kcal/mol), Met165 (−3.9 kcal/mol), and Leu167 (−3.4 kcal/mol). This result can be confirmed from the ES and CT+mix components of PIEDA (Figures S4d, S4l, and S5h); the carbonyl oxygen atom on the 2-pyrrolidone of 9I7(4) and the hydrogen atom attached to the nitrogen atom on the imidazole ring in the side chain of His163 were NH–O hydrogen-bonded. In addition, the hydrogen atom attached to the nitrogen atom on the 2-pyrrolidone of 9I7(4) and the oxygen atom on the carboxylic acid in the side chain of Glu166 were also NH–O hydrogen-bonded. From the DI component of PIEDA (Figures S4p and S5h), it was inferred that the hydrogen atom attached to the carbon atoms on the alkyl group of 2-pyrrolidone and the π-orbitals on the amide groups of the amino acid residues around the inhibitor (e.g., the main chain between Met165 and Glu166 (DI: −6.2 kcal/mol) and the side chain of Asn142 (DI: −2.6 kcal/mol)) exhibited CH/π interactions.

In summary, IFIE/PIEDA analysis with ligand fragmentation enabled us to identify the critical amino acid residues for the binding of nirmatrelvir to SARS-CoV-2 M^pro^ and their types of interactions on a functional group of the ligand.

### 3.2 Accuracy Verification of FMO Calculation for Zn-Containing Proteins Using the Fragment Merge Function

**Figure 3:**
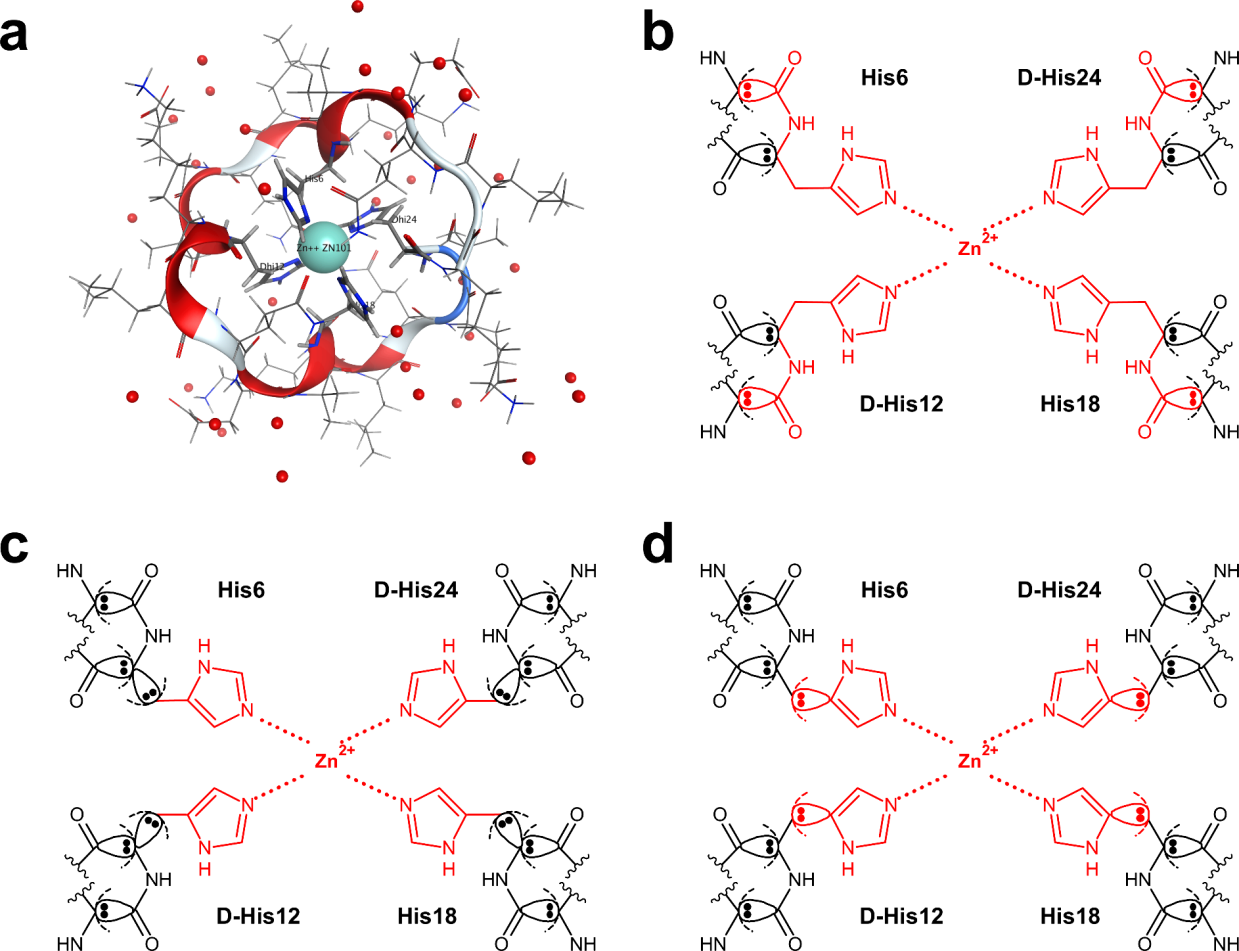
Fragmentation around Zn^2+^ of a cyclic peptide coordinated with the Zn^2+^ ion. The three-dimensional structure of the cyclic peptide (PDB ID: 6UFA) is illustrated (a). The Zn^2+^ ion is a CPK model, and the water molecules are a ball model. The four histidines coordinated with the Zn^2+^ ion of the cyclic peptide are represented by stick models, and other amino acid residues are represented by line models. Three fragmentation schemes are depicted in the main chain fragmentation (b), the main/side chain (CB–CA) fragmentation (c), and the main/side chain (CB–CG) fragmentation (d). The atoms colored red are merged into the Zn^2+^ fragment.

An example in which fragmentation processing is complicated is a molecular system containing a transition metal ion (e.g., Zn^2+^ and Mg^2+^) or an ion (e.g., Ca^+^ and Na^+^). For example, the structure of the 6UFA entry^70^ registered in the PDB contains a Zn^2+^ ion at the center of the cyclic peptide, where the Zn^2+^ ion forms a coordination bond with the four surrounding histidines: His6, d-His12, His18, and d-His24 (Figure 3a). The cyclic peptide has the sequence KLqeXHklQEXhKLqeXHklQEXh, where X represents α-aminoisobutyric acid (AIB) and upper- and lower-case letters indicate l- and d-amino acids, respectively. Treating a Zn^2+^ ion as a single fragment worsens the self-consistent field convergence of monomers of the ion, the amino acid residue fragments coordinated with it, and dimers containing the ion.^62^ This is because the proper electronic state cannot be solved, as the coordination bond is broken. Thus, treating the Zn^2+^ ion and the histidines in the vicinity coordinated with the ion as one fragment is necessary to avoid breaking the coordination bond. However, a large fragment will be formed if this merging process is performed on a fragment of a typical amino acid residue unit;^62^ this fragmentation scheme is referred to here as “main chain fragmentation” (Figure 3b). The total computational cost of a whole protein increases even if one large fragment is included. Therefore, it is practical to reduce the cost of calculation by dividing the histidine fragment coordinated with the Zn^2+^ ions into the main and side chains and merging only the side chain with the Zn^2+^ ions (Figures 3c and 3d); the main/side chain (CB–CA) fragmentation, as seen in Figure 3c, is a method that has often been performed in the past,^61,62,64^ while the main/side chain (CB–CG) fragmentation, as seen in Figure 3d, is the first attempt to validate the accuracy of metalloprotein fragmentation. However, because this fragmentation process is very complicated to handle manually, we implemented the merge function to be efficiently executed by FMOe (described in Section S1.2.2).

First, the structure was pretreated using the following procedure. This cyclic peptide structure (PDB ID: 6UFA) is a high-resolution X-ray crystal structure (0.77 Å resolution). The structure had no missing residues, and most of the hydrogen atoms for the cyclic peptide molecule were registered. However, hydrogen atoms are not present in water molecules. Therefore, the hydrogen atoms on the cyclic peptide were used as is, whereas only water molecules were hydrogenated using the AddH function of the MOE. Next, structural optimization was performed with the Amber10:EHT force field using energy minimization via the MOE. The structure was highly reliable because these data were obtained using high-resolution X-ray crystallography. Therefore, the atoms in the molecular system were constrained during structure optimization under the following conditions. All atoms registered in the PDB were fixed, and only the complementary hydrogen atoms on the water molecules were unconstrained.

The FMO calculation times, total energies of the whole molecular system, PIEDA of the Zn^2+^ ion fragment, atomic charges of the Zn^2+^ ion and the four histidines, His6 (HIS6), His18 (HIS18), d-His12 (DHI12), and d-His24 (DHI24), coordinated with the Zn^2+^ ion, and the FMO-based electron densities were investigated to verify the calculation efficiency and accuracy of the Zn^2+^ ion fragmentation schemes. Table S2 lists the FMO calculation times for the three fragmentations (Figures 3b–3d). By fragmentation between the main and side chains, it can be confirmed that the calculation time was significantly reduced to 1/6 and 1/10 for the MP2/6-31G and MP2/6-31G* calculations, respectively, compared to the main chain fragmentation. In addition, the result of the FMO calculation for the main chain fragmentation, which has fewer fragmentation points, was used as a reference value for a high-precision calculation when verifying the accuracy of the physical quantities of the two side-chain fragmentations. In Table S2, the total energies of the main/side chain fragmentation schemes are compared with the results of the main-chain fragmentation. As a result, at each calculation level (HF and MP2 methods), we verified that the differences from the total energies from the main chain fragmentation were less than 0.90 and 0.36 hartree in the main/side chain (CB–CA) and main/side chain (CB–CG) fragmentations, respectively.

**Figure 4:**
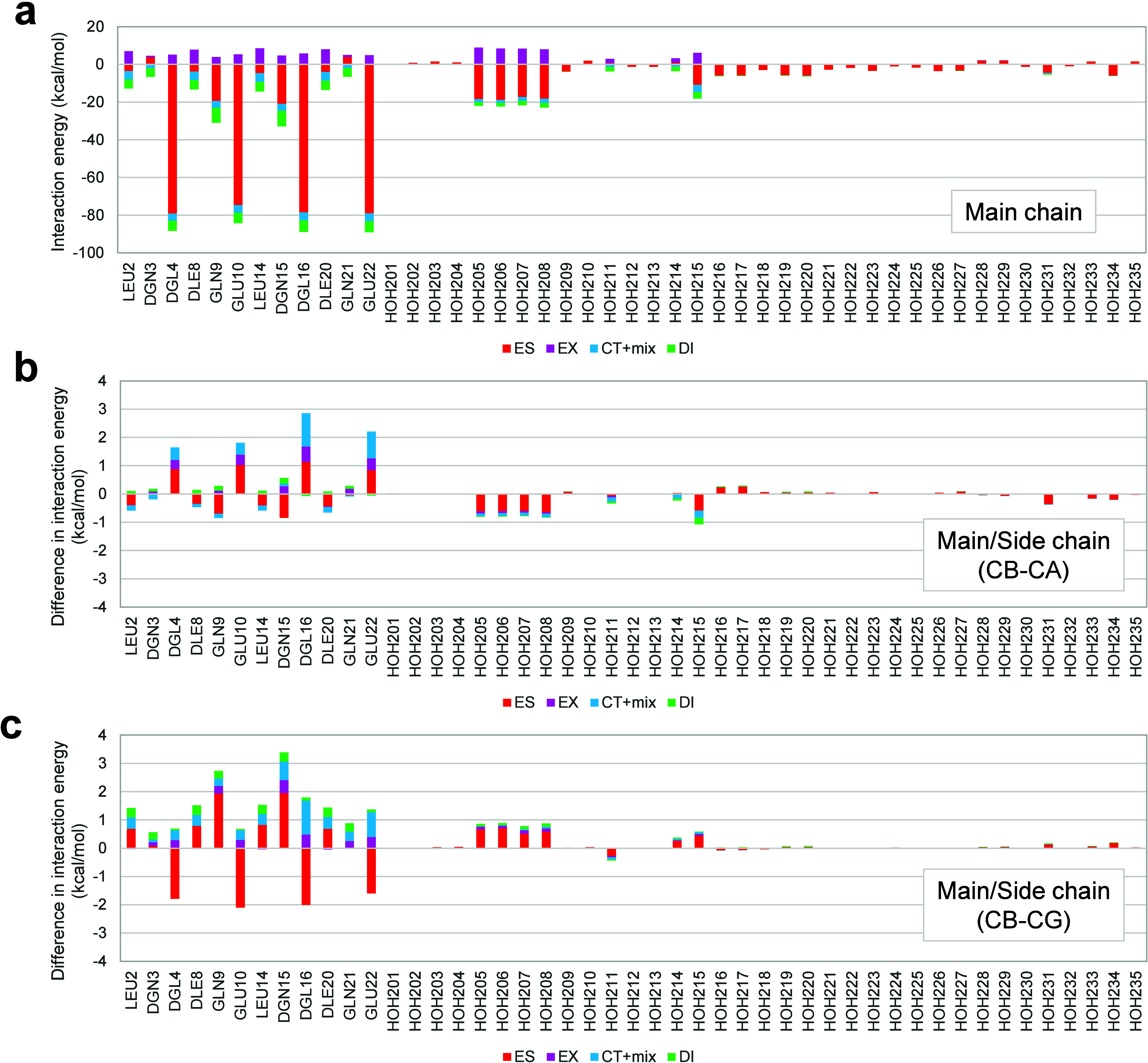
Interaction energy analysis of Zn^2+^ ion fragment, including the four histidines for the cyclic peptide containing Zn^2+^ (PDB ID: 6UFA). Interaction energies with PIEDA for main chain fragmentation data (FMODB ID: 8NN1Y) are shown in (a). Differences in interaction energies between the main chain fragmentation data and the main-side chain (CB–CA) fragmentation (FMODB ID: GYYM1) and the main/side chain (CB–CG) fragmentation (FMODB ID: 166QZ) are shown in parts (b) and (c), respectively. The fragment units in the interaction analysis were adjusted to the main chain fragmentation scheme to compare the data to the main chain fragmentation data.

As seen in Figures 4 and S6, interaction energy analysis with PIEDA of the Zn^2+^ ion fragment containing the four histidines was performed in the main chain fragmentation units to examine the accuracy of the fragmentation schemes in terms of interactions. Figures 4a, S6a, and S6b show that the qualitative trends of IFIE/PIEDA were consistent with the main chain fragmentation and the main/side chain fragmentations. As shown in Figures 4b and 4c, the differences in interaction energy from the main chain fragmentation to the two main/side chain fragmentation data sets were within ca. 3 kcal/mol. Next, from the FMO calculation results of the main/side chain (CB–CA) and main/side chain (CB–CG) fragmentations, Figure S7 shows the PIEDA between the fragments containing Zn^2+^ ions and each of the other fragments. The trends of the ES, EX, CT+mix, and DI components were almost identical for both fragmentation schemes. However, there was an exception in the CT+mix component of the four fragments, which showed a strong repulsive interaction of approximately +100 kcal/mol in the main/side chain (CB–CA) fragmentation. Here, we focused on these fragment pairs for the following reasons: The bare CT interaction energy should have negative values in nature. In fact, statistical analysis of IFIE/PIEDA for FMODB confirmed that for fragment pairs forming typical interactions such as hydrogen bonds, XH/π, and ion pairs, the CT+mix terms tend to be essentially attractive interaction energies in our previous work.^28,72^ The CT+mix values between the four fragments and the Zn^2+^ ion fragment obviously differ from the tendency. The four fragments were the amino acid residues (Lys1, d-Lys7 (DLY7), Lys13, and d-Lys19 (DLY19)) immediately after the histidine that were merged into the Zn^2+^ ion fragment. Notably, the CT+mix components of the four fragments in the main/side chain (CB–CA) fragmentation showed CT+mix components of +100 kcal/mol. In contrast, the CT+mix components of the four fragments in the main/side chain (CB–CG) fragmentation were +30 kcal/mol, and the repulsive interactions were weaker than those in the main/side chain (CB–CA) fragmentation. These repulsions of the CT+mix components affect the accuracy of the total energy. This outcome may be due to the proximity of the BDAs of the CA carbon atom in the main chain and the CB carbon atom in the side chain. Therefore, it is recommended to exclude these fragment pairs from the IFIE/PIEDA analysis in the main/side chain fragmentation. This recommendation is because these fragment pairs show unusual values of the CT+mix terms due to fragment pairs with the BDAs next to each other. Similarly, in the conventional IFIE analysis of protein, IFIE values between covalent bond fragments, i.e., fragment pairs in which the BDA is next to the BAA, are excluded from the analysis due to unusual IFIE values caused by bond detachment in the FMO calculation.^73,74^

Tables S3 and S4 summarize the atomic charges of the Zn^2+^ ion and the four histidines, His6 (HIS6), His18 (HIS18), d-His12 (DHI12), and d-His24 (DHI24), based on the FMO calculation results (HF/6-31G and HF/6-31G* levels). Note that the atomic charge trends between the data using the 6-31G and 6-31G* basis functions were consistent. Figures S8–S10 show the net atomic charges for three fragmentation schemes and the differences in the atomic charges between the main chain fragmentation and the main/side chain fragmentation data. As shown in Table S4 and Figure S8 and S9, the trends of the Mulliken and natural population analysis charges were consistent regardless of the fragmentation method. Moreover, most of the atoms have a difference of ca. 0.05*e* or less compared to the atomic charge of the main-chain fragmentation method. As an exception, the C, CB, CG, and CD2 carbon atoms of each histidine in the main/side chain (CB–CA) fragmentation and the CA, C, CB, and CD2 carbon atoms of each histidine in the main/side chain (CB–CG) fragmentation had a difference of ca. 0.05*e* or more. They were the BDAs, BAAs, or their neighbors. In contrast, the value of the Merz–Kollman charge varied significantly depending on the fragmentation method from Table S4 and Figure S10. It was inferred that this outcome was due to the fitting performed to reproduce the electrostatic potential and the difference in the atoms constituting the fragment. Therefore, in the future, when assigning an atomic charge using an electrostatic potential charge (e.g., RESP or Merz–Kollman) to construct an FMO force field, careful attention should be paid to the fragmentation treatment of the metal-containing proteins.

Finally, the FMO-based electron density was analyzed to verify that the electronic structure calculations were accurate. Figures S11a–S11c show the FMO-based electron densities ρ of the cyclic peptides obtained using the main chain fragmentation scheme; Figures S11d–S11i show the differences in the electron density Δρ between the main chain and the main/side chain fragmentation data. We compared the FMO-based electron density of the main chain fragmentation with that of the main/side chain fragmentation using Δρ. At a high electron density level (ρ and Δρ = 0.05*e*), there was no significant difference in electron density around the Zn^2+^ ion between the main chain and the two main/side chain fragmentations (Figure S11d and S11g). At low levels of electron density (ρ and Δρ = 0.01e or 0.005*e*), slight and or significant differences were observed in the electron density between the main chain and main/side chain fragmentations around the BDAs and BAAs of the four histidines (Figures S11e, S11f, S11h, and S11i). The main/side chain (CB–CG) fragmentation differed less from the main/side chain (CB–CA) fragmentation. In the case of the main/side chain (CG–CA) fragmentation, the spatial variation in electron density was more widely affected than that of the main/side chain (CB–CA) fragmentation. This result is consistent with the atomic charge analysis data, and the change in the electron density distribution explains the differences in the atomic charges of the imidazole ring of the histidine. Therefore, main/side chain (CB–CG) fragmentation was confirmed to be the calculation method with the fewest splitting errors, even in terms of electron density.

From the above results, the accuracy verification of the total energy, IFIE, atomic charge, and electron density compared with the calculation efficiency and main chain fragmentation in this molecular system indicates that main/side chain (CB–CG) fragmentation is an appropriate fragmentation method. However, metal-containing proteins have variations in the amino acid residues coordinated with the metal and in their sites (main chain and side chain). Therefore, the fragmentation of the main/side chain (CB–CG) is not always a general-purpose method. In particular, the electronic state of a metal is essential in a reaction; therefore, it is necessary to develop a fragmentation method that can adequately handle the electronic state. In the future, we will accumulate FMO calculation examples for metal-containing proteins and aim to prepare rules for the appropriate fragmentation methods.

We believe that these findings will be useful not only in FMO calculations but also in examining the molecular division patterns of ligand- and metal-containing biomolecular systems using other fragmentation methods.^1,2,75,76^

## 4 Conclusion

In this study, we reported a library with an MOE for creating input files for FMO calculations performed using ABINIT-MP and analyzed the results. This approach makes it possible to complete the construction of molecular structures, generate input files for FMO calculations, and explore the results. As an example of using FMOe, we performed IFIE/PIEDA interaction analysis by ligand fragmentation for the complex between the tripeptide inhibitor nirmatrelvir, with a Cys145 covalent bond, and SARS-CoV-2 M^pro^. This example is challenging to handle using the current automatic fragmentation function of ABINIT-MP. Nevertheless, using FMOe, we performed FMO calculations relatively easily and identified the key amino acid residues of M^pro^ in nirmatrelvir binding and their interactions with the functional groups of the inhibitor. Next, the accuracy of the fragmentation method for Zn^2+^ ions was verified for a cyclic peptide coordinated with a Zn^2+^ ion. Here, we attempted the following fragmentations: the four histidines, His6, His18, d-His12, and d-His24, coordinated with the Zn^2+^ ion were divided into the main chain and the side chain with the BDA at the CB carbon atom and the BAA at the CA or CG carbon atom, and the side chains of histidines and the Zn^2+^ ion were merged into one fragment. Consequently, main/side chain (CB–CG) fragmentation was calculated efficiently and accurately.

FMOe simplifies complicated fragmentation, such as that of covalent inhibitors, and is also expected to efficiently handle medium-molecular-weight drug candidate compounds for diversifying drug discovery modalities (e.g., cyclic peptides). In addition, the examination of metal-containing proteins, where an amino acid residue is coordinated with a metal (e.g., an enzyme), was accelerated. In the future, it is expected that the accumulation of knowledge of the FMO calculation results of these complicated molecular systems will lead to the establishment of general-purpose fragmentation rules that enable more convenient automatic fragmentation processing. Therefore, we plan to simplify the workflow and consequently accelerate the research.

## Data Availability

The source code of FMOe is available from https://github.com/drugdesign/FMOE. The FMO calculation data is available from https://drugdesign.riken.jp/FMODB/.

## Supporting Information Available

Section S1: Methods. Section S2: Details of IFIE/PIEDA analysis between SARS-CoV-2 M^pro^ and nirmatrelvir. Section S3: Details of the accuracy verification of the fragmentation schema for four histidines coordinated with the Zn^2+^ ion (PDF)
