# Supplementary material for: FMOe: Preprocessing and Visualizing Package of the Fragment Molecular Orbital Method for Molecular Operating Environment and Its Applications in Covalent Ligand and Metalloprotein Analyses
Source: J Chem Inf Model. 2024 Sep 5;64(18):6927–37. doi: 10.1021/acs.jcim.4c01169 (PMC11505893; doi:10.1021/acs.jcim.4c01169)
Supplement: Supplementary file 1 — ci4c01169_si_001.pdf [file ci4c01169_si_001.pdf]

Supporting information

FMOe: Preprocessing and Visualizing Package of the Fragment Molecular Orbital Method for Molecular Operating Environment and Its Applications in Covalent Ligand and Metalloprotein Analyses

Hirotomo Moriwaki[1,#], *Yusuke Kawashima*[2,#], *Chiduru Watanabe*[1,3,*], *Kikuko Kamisaka*[1], *Yoshio Okiyama*[4], *Kaori Fukuzawa*[2,5], *Teruki Honma*[1]

[1]Center for Biosystems Dynamics Research, RIKEN,

1-7-22 Suehiro-cho, Tsurumi-ku, Yokohama, Kanagawa 230-0045, Japan

[2]Department of Physical Chemistry, School of Pharmacy and Pharmaceutical Sciences,

Hoshi University, 2-4-41 Ebara, Shinagawa-ku, Tokyo 142-8501, Japan

[3]JST PRESTO, 4-1-8, Honcho, Kawaguchi, Saitama 332-0012, Japan

[4]Department of Computational Science, Graduate School of System Informatics,

Kobe University, 1-1 Rokkodai, Nada-ku, Kobe, Hyogo 657-8501, Japan

[5] Graduate School of Pharmaceutical Sciences, Osaka University,

1-6 Yamadaoka, Suita, Osaka 565-0871, Japan

[#]equal contributions

*corresponding author

*Chiduru Watanabe (E-mail: chiduru.watanabe@riken.jp)

Table of contents

S1. Methods 3

S1.1. Fragment molecular orbital (FMO) method 3

S1.2. Functions and operating procedures of FMOe 5

S1.2.1. Preprocessing function: automatic and manual fragmentations and input file generation 5

S1.2.2. Preprocessing function: automatic, manual, and merge fragmentations and input file generation 8

S1.2.3. Postprocessing function: IFIE/PIEDA analysis 11

S2. Details of IFIE/PIEDA analysis between SARS-CoV-2 M^{pro} and nirmatrelvir 14

S3. Details of the accuracy verification of the fragmentation schema for four histidines coordinated with the Zn^{2+} ion 16

S1. Methods

S1.1. Fragment molecular orbital (FMO) method

Herein, we briefly describe the *ab initio* FMO method[1]. In this method, a large molecule or molecular cluster is divided into small fragments, and MO calculations are performed on each monomer and dimer fragment, thus yielding the properties of the entire system. The many-body effects were considered using the environmental electrostatic potentials. The total energies of the FMO calculations are given by Equation (S1).

$$E_{\mathrm{total}} = \sum_{I} E'_{I} + \sum_{I<J} \Delta\tilde{E}_{IJ}. \qquad \text{(S1)}$$

Here, E'_{I} is the monomer energy without the environmental electrostatic potential; $\Delta\tilde{E}_{IJ}$ is the interfragment interaction energy (IFIE); and I and J are fragment indices. In addition, using the Møller–Plesset perturbation method[2], pair interaction energy decomposition analysis (PIEDA)[3–5], was used to analyze the energy components of IFIE, $\Delta\tilde{E}_{IJ}$: the electrostatic interaction (ES), exchange repulsion (EX), charge transfer interaction with mixing terms (CT+mix), and dispersion interaction (DI), as shown in Equation (S2).

$$\Delta\tilde{E}_{IJ} = \Delta\tilde{E}_{IJ}^{\mathrm{ES}} + \Delta E_{IJ}^{\mathrm{EX}} + \Delta E_{IJ}^{\mathrm{CT+mix}} + \Delta E_{IJ}^{\mathrm{DI}}. \qquad \text{(S2)}$$

To obtain interaction energy of several fragments for A part $\Delta\tilde{E}_{J}^{\mathrm{A}}$, such as the inhibitor-binding energy, we summed the IFIEs of all pairs between the fragments for A part and a fragment J that called the "IFIE-sum"[6] and is given by the IFIEs shown in Equation (S3).

$$\Delta\tilde{E}_{J}^{\mathrm{A}} = \sum_{I=\mathrm{A}} \Delta\tilde{E}_{IJ}. \qquad \text{(S3)}$$

The electron densities of the FMO calculations[7], $\rho(\boldsymbol{r})$, are given by Equation (S4).

$$\rho(\boldsymbol{r}) = \sum_{I} \rho_I(\boldsymbol{r}) + \sum_{I<J} \Delta\rho_{IJ}(\boldsymbol{r}). \qquad \text{(S4)}$$

Here, ρ_I is the monomer electron density without the environmental electrostatic potential and $\Delta\rho_{IJ}$ is the dimer electron density.

Table S1 lists the pre/postprocessing graphical user interfaces (GUIs)[8–19] of FMO processing programs[20–28]. In this study, the details of the FMO calculation by ABINIT-MP and FMOe, such as structural preprocessing and fragmentation, are described in Sections 3.1 and *S1.2.1* for a complex between the SARS-CoV-2 main protease (M^{pro}) and a tripeptide-like inhibitor, nirmatrelvir (brand name: Paxlovid, code name: PF-07321332)[29,30] covalently bonded with Cys145 of M^{pro} and Sections 3.2 and *S1.2.2* for a cyclic peptide with a Zn^{2+} ion as the structural center[31].

Table S1. List of graphical user interfaces (GUIs) for FMO processing programs[20–28].

GUIs	FMO processing programs	Functions
FU[8,9]	^{a}GAMESS	Pre/postprocessing
FACIO[10–12]	^{a}GAMESS	Pre/postprocessing
MOE-FMOutil[13]	^{a}GAMESS	Pre/postprocessing
BioStation Viewer[14,26,32]	^{b}ABINIT-MP	Pre/postprocessing
FMOe[15]	^{b}ABINIT-MP	Pre/postprocessing
View PIEDA[16]	^{b}ABINIT-MP	Postprocessing
PAICSView[17,28]	^{c}PAICS	Pre/postprocessing
HyperChem[18]	^{a}GAMESS, ^{b}ABINIT-MP, ^{c}PAICS	Pre/postprocessing
AnalysisFMO[19]	^{a}GAMESS, ^{b}ABINIT-MP, ^{c}PAICS	Postprocessing

^{a}The general atomic and molecular electronic structure systems (GAMESS)[20–23]
^{b}The *ab initio* fragment MO method programs (ABINIT-MP)[24–26]
^{c}The parallelized *ab initio* calculation systems (PAICS)[27,28]

S1.2. Functions and operating procedures of FMOe

S1.2.1. Preprocessing function: automatic and manual fragmentations and input file generation

Figure S1 shows the interface for fragmentation. For structures that have undergone hydrogenation and structural optimization (i.e., structures in which the bond order between atoms is appropriately set on the MOE and the force field and partial charge are assigned), fragmentation can be performed using the following procedure:

When selecting the "*Fragmentation/Merge*" mode from the "*FMOe*" button (Figure S1a) on the right side of the MOE's primary interface window ("*MOE*" window), the "*FMOe Fragmentation*" window (Figure S1b) was launched. Fragmentation of the protein was performed automatically in units of amino acid residues. Each water molecule, ligand, and metal ion was treated as one fragment. In Figure S1a, the fragment points to be automatically divided are displayed on the viewer as a white disk, considering the orientation of BDA and BAA. A small white stud was observed on the side of the BAA. The functions used for fragmenting were also designed to be used in SVL. Therefore, automatic processing can be performed.

The covalent bond serial number to be divided (#), bond split types of automatic or manual fragmentation (T), and information on BDA and BAA are displayed in the "*Bonds*" table in the "*FMOe Fragmentation*" window (Figure S1b). Selecting the division site from the list allows the user to zoom into the site and delete the site between the BDA and BAA.

The serial number of the fragment to be divided (#), residue name (Res.), number of atoms in each fragment (nA), number of atomic orbitals (AO) in the selected basis set (default: 6-31G*), and several types of charge

information (D: number of detached electron pairs, A: number of attached electron pairs, and C: fragment formal charge) are displayed in the “*Fragments*” table of the “*FMOe Fragmentation*” window (Figure S1b). By clicking on each fragment in the “*Fragments*” table, all atoms in the fragment can be selected in the “*MOE*” window.

The details of manual fragmentation are demonstrated in the SARS-CoV-2 M^{pro} inhibitor, nirmatrelvir, analysis example described in Section 3.1. Suppose additional fragmentation, such as ligand fragmentation in the functional group unit, is desired. In that case, the following procedure can be used (Figures S1b and S1c): After clicking the “+” button, meaning the “*Manual Fragmentation*” (Figure S1b), the covalent bond can be divided manually by clicking the BDA and BAA in sequence (Figure S1c). If the sp^3 carbon atom used as the BDA was selected, the atom that was a candidate for BAA is displayed as a ball. A light blue disk represents the fragment point between the BDA and BAA to be manually divided, where a small light blue stud is considered the BAA side.

After performing the fragmentation process correctly, the FMO calculation input file was generated for ABINIT-MP using the following procedure: When clicking the “*Generate*” button (Figure S1b) at the bottom of the “*FMOe Fragmentation*” window, the molecule coordinate file (protein data bank (PDB)), which was required for the FMO calculation, and ABINIT-MP input file (the “ajf file”) were generated. In this process, one can not specify the FMO configuration, including the computational method and basis set. However, it is possible to reflect the settings in the ajf file in production by loading a user-defined ajf template containing

those settings. Therefore, it is possible to deal flexibly with the incompatibilities of configuration files due to differences in the ABINIT-MP versions.

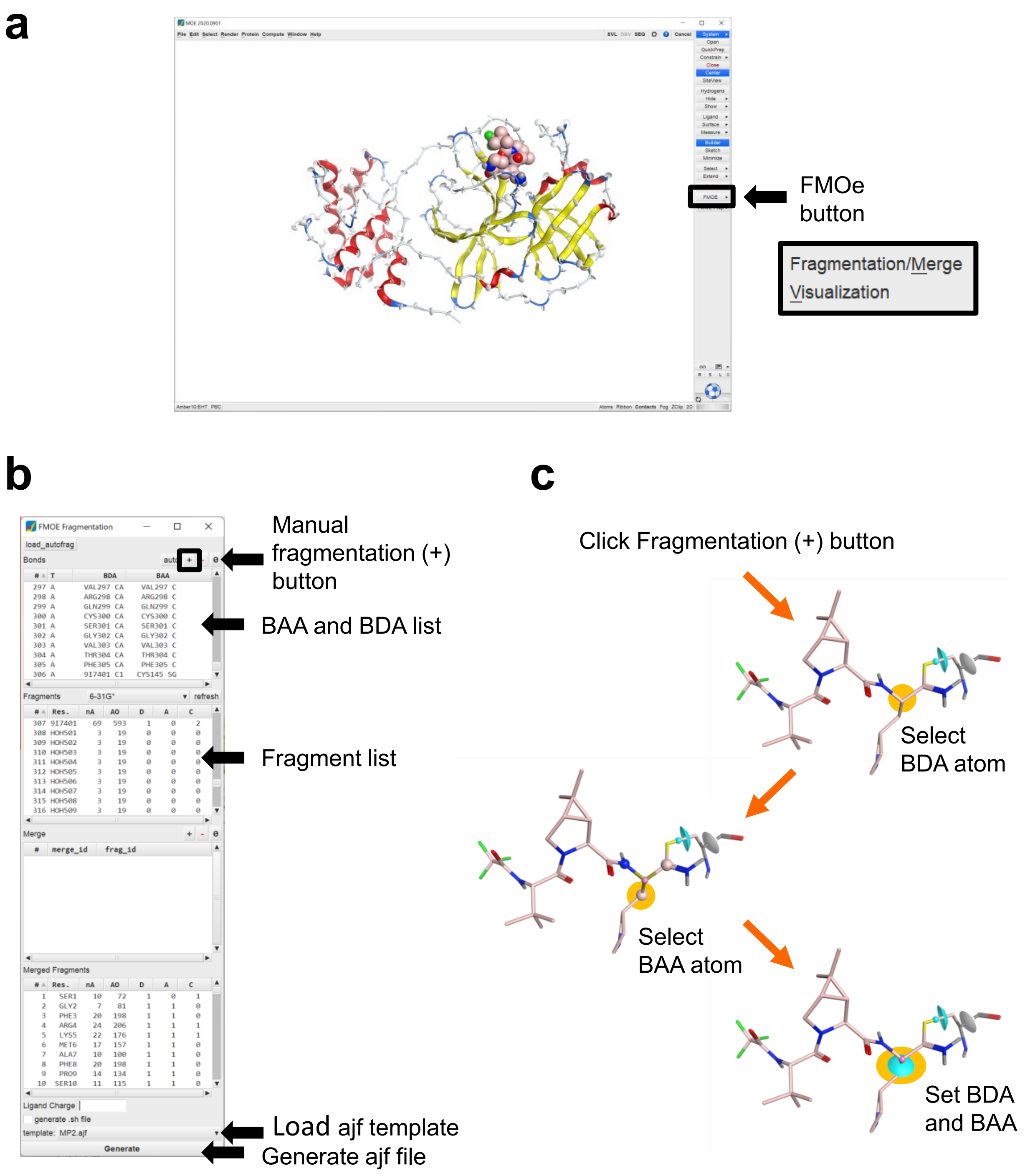

Figure S1. Manual fragmentation interface by the "*Fragmentation/Merge*" mode of FMOe. The "*FMOe*" start button and the "*MOE*" window after automatic fragmentation processing (a), "*FMOe Fragmentation*" window (b), and manual fragmentation processing procedure (c) are shown. The structure used was that of a SARS-CoV-2 M^{pro} and nirmatrelvir complex (PDB ID: 7SI9).

S1.2.2. Preprocessing function: automatic, manual, and merge fragmentations and input file generation

Merging multiple fragments was possible using the "*Merge*" table in the "*FMOe Fragmentation*" window even for fragments once divided. The details of the fragment merge function are introduced in the metalloprotein analysis example described in Section 3.2. For the cyclic peptide with Zn^{2+} ion after structure optimization, fragmentation of the protein was performed automatically in units of amino acid residues by the "*Fragmentation/Merge*" mode of FMOe (Figures S1a and S2a). Next, side-chain fragmentation of histidine coordinated with Zn^{2+} ions (Figures 3c and 3d) was performed according to the manual fragmentation procedure described in Section *S1.2.1*. Subsequently, for the fragment to be merged, at least one atom was selected from each fragment, comprising the five fragments containing Zn^{2+} and four side chains of the histidines (Figure S2b). Finally, by pressing the "+" button, meaning the "*Merge Fragment*," on the right side of the "*Merge*" table in the "*FMOe Fragmentation*" window, the fragments containing the selected atoms were merged into one fragment (Figure S2b). Fragment information after merging can be found in the "*Merged Fragments*" table in the "*FMOe Fragmentation*" window (Figure S2c). The atomic details comprising the fragment can be confirmed using the following procedure. When the relevant fragment in the "*Merged Fragments*" table was selected, atoms that make up the fragment were selected on the molecular structure of the "*MOE*" window, and the atomic information of the fragment could be obtained (Figure S2c). A similar procedure can merge the Zn^{2+} ion and four histidines, His6 (HIS6), His18 (HIS18), D-His12 (DHI12), and D-His24 (DHI24), after main-chain fragmentation (Figure 3b). Once the fragment merge was complete, a set of

FMO calculation input files was created by clicking the "*Generate*" button at the bottom of the "*FMOe Fragmentation*" window (Figure S2c). The FMO-MP2/6-31G and FMO-MP2/6-31G* calculations were performed using the obtained FMO calculation input file. The results of the FMO calculation at MP2/6-31G for the main chain, main/side chain (CB–CA), and main/side chain (CB–CG) fragmentations (Figures 3b, 3c, and 3d, respectively) were registered in FMODB[33,34] with the codes (FMODB ID) VKK41, YNN22, and 5NRZZ, respectively; those at MP2/6-31G* for the main chain, main/side chain (CB–CA), and main/side chain (CB–CG) fragmentation were registered in FMODB[33,34] with the codes (FMODB ID) 5NRZZ, GYYM1, and 166QZ, respectively.

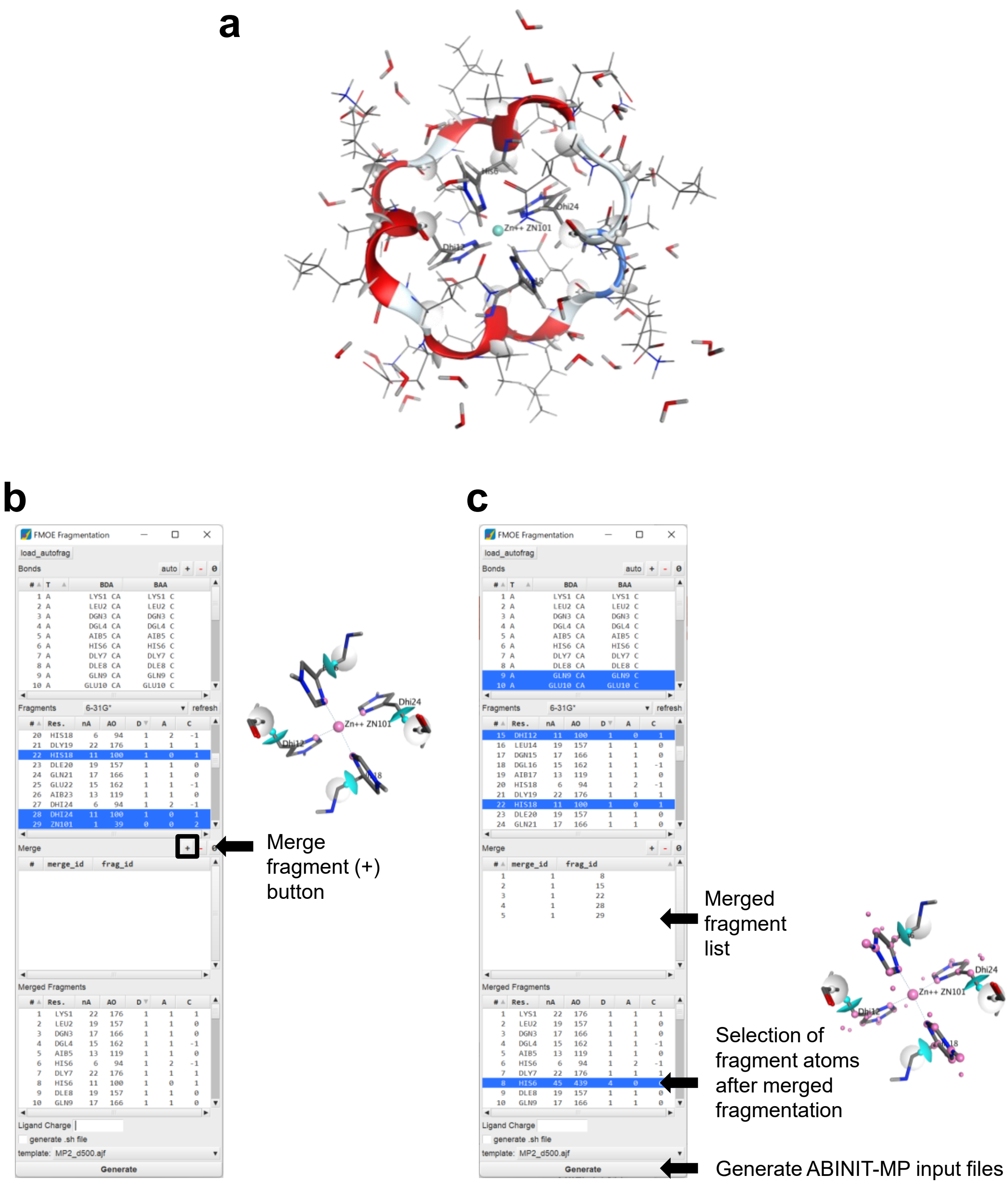

Figure S2. Merge fragment flow. Automatic fragmentation around Zn^{2+} ion of a cyclic peptide coordinated with the transition metal (PDB ID: 6UFA) is illustrated (a). The Zn^{2+} ion is a ball model, and the water molecules are a stick model. The four histidines coordinated with the Zn^{2+} of the cyclic peptide are represented by a stick model, and other amino acid residues are represented by line models. After automatic fragmentation, the atoms contained in the fragments to be merged are selected, and the "+" button, meaning the "*Merge Fragment*," in the "*FMOe Fragmentation*" window is clicked (b). The "*Merge*" table in the "*FMOe Fragmentation*" window and the "*Merged Fragments*" table reflect the fragment information after fragment merging (c).

S1.2.3. Postprocessing function: IFIE/PIEDA analysis

Figure S3 shows the visualization interface for the results of FMO calculations. Postprocessing for the IFIE/PIEDA analysis was performed as follows: The "*Visualization*" mode (Figure S1a) was selected using the "*FMOe*" button on the right side of the "*MOE*" window to read a checkpoint file (CPF). The CPF file describes the FMO calculation results executed using ABINIT-MP. The results of the FMO calculation were handled with the CPF file for visualization and were read by both FMOe and BioStation Viewer. IFIE/PIEDA analysis was performed automatically (Figure S3a). The fragment to be analyzed was set to the ligand molecule by default. The fragment to be analyzed; components of interaction energies, second-order Møller–Plesset perturbation theory (MP2), Hartree–Fock (HF), electrostatic interaction (ES), exchange repulsion (EX), charge transfer with mixing terms (CT+mix), and dispersion interaction (DI) to be visualized; and the color scale range of the interaction energy was specified in the "*FMO Visualization*" window (Figure S3b). These operations immediately reflected the coloring of the interaction energy value of the selected component on the molecule in the viewer (Figure S3a). To change the fragment to be analyzed, at least one atom of the target fragment was selected in the "*MOE*" window. Then, the "*Set*" button, meaning the "*Set Fragment*," was selected in the "*FMO Visualization*" window to reflect the results of the IFIE/PIEDA analysis of the target fragment. The IFIE or IFIE-sum of the target fragment with the fragment(s) selected on the screen is displayed in the upper left of the "*MOE*" window (Figure S3c) and is also shown in the "*SVL Commands*" window

(Figure S3d). The "*List*" button (Figure S3b) can be pressed at the top right of the "*FMO Visualization*" window to display a list of IFIE/PIEDA for the target fragment on the screen (Figure S3e).

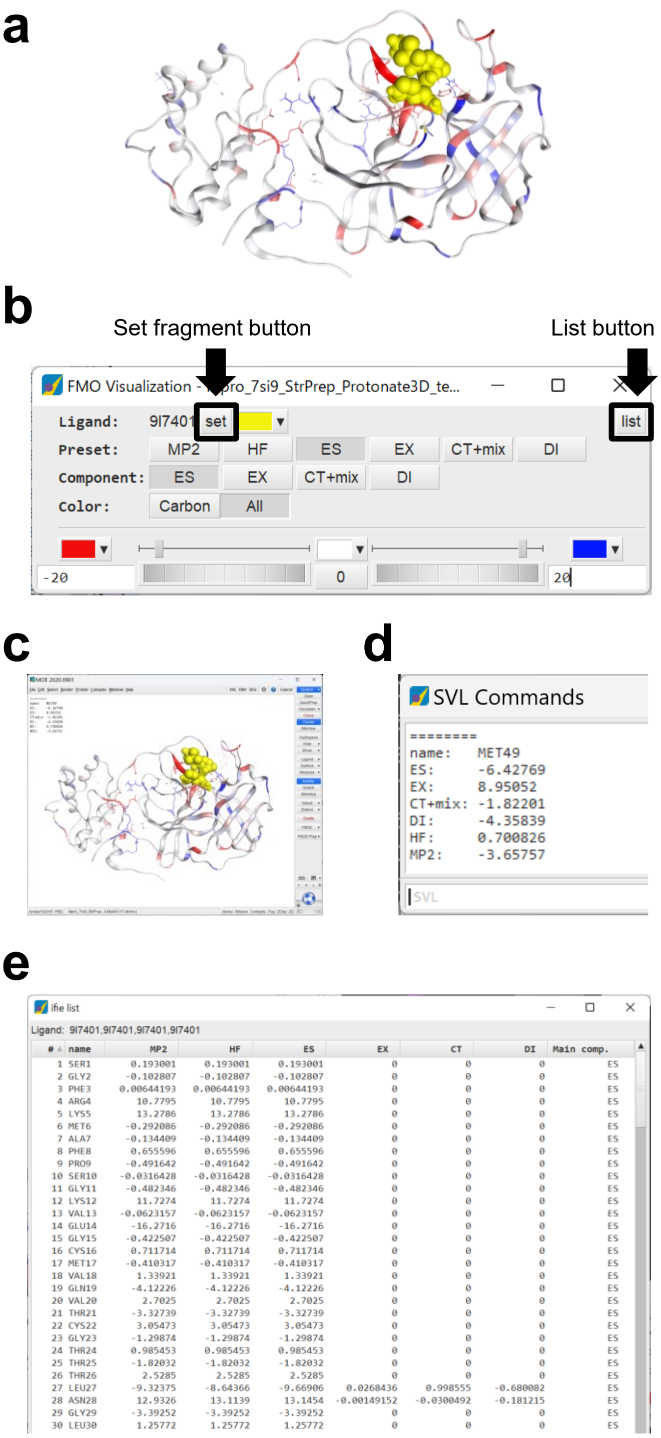

Figure S3. Interfragment interaction energy (IFIE) analysis and pair interaction energy decomposition analysis (PIEDA) interface by the "*Visualization*" mode of FMOe. The IFIE (MP2) values (attractive interaction: red, repulsive interaction: blue) of the fragment (yellow) selected for interaction analysis and the fragment of the exception are mapped on the molecular structure (a); the "*FMO Visualization*"

window allows for the specification of IFIE/PIEDA components and color scales (b); the IFIE or IFIE-sum of the target fragment (yellow) with the fragment(s) selected on the screen is displayed in the upper left of the "*MOE*" window (c) and is also shown in the "*SVL Commands*" window (d); and the IFIE/PIEDA list can be obtained from the "*List*" button in the "*FMO Visualization*" window (e). For the FMO data used, the FMO calculation results of the SARS-CoV-2 M^{pro} and nirmatrelvir complex (PDB ID: 7SI9; FMODB ID: 4LQRN) were used.

S2. Details of IFIE/PIEDA analysis between SARS-CoV-2 M^{pro} and nirmatrelvir

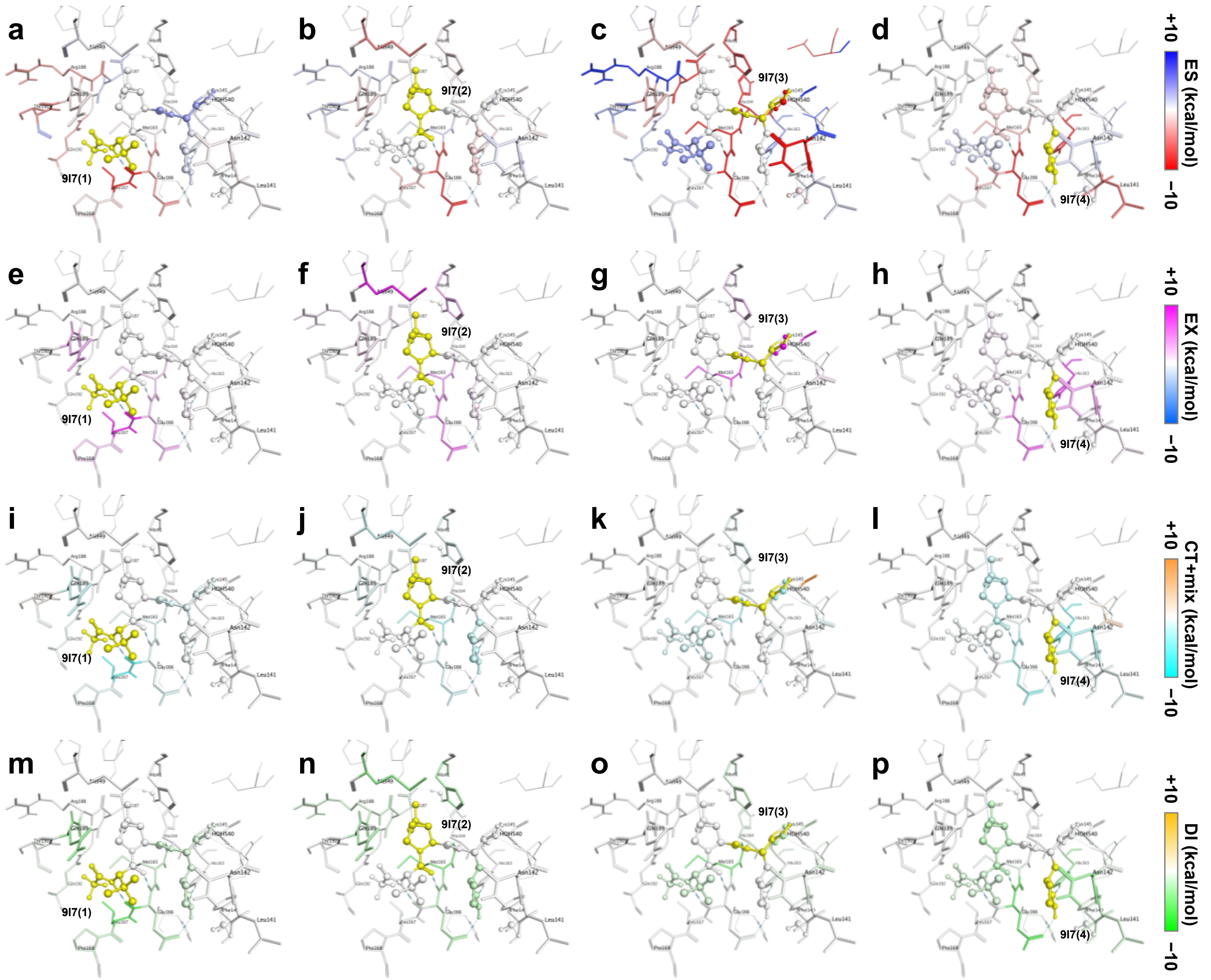

Figure S4. Pair interaction energy decomposition analysis (PIEDA) between nirmatrelvir (9I7) and each amino acid residue of SARS-CoV-2 M^{pro} using ligand fragmentation. Electrostatic (ES), exchange repulsion (EX), charge transfer interaction with mixing terms (CT+mix), and dispersion interaction (DI) energy analyses of 9I7(1) fragment with each amino acid (a), (b), (c), and (d), respectively. ES, EX, CT+mix, and DI energy analyses of 9I7(2) fragment with each amino acid (e), (f), (g), and (h), respectively. ES, EX, CT+mix, and DI energy analyses of 9I7(3) fragment with each amino acid (i), (j), (k), and (l), respectively. ES, EX, CT+mix, and DI energy analyses of 9I7(4) fragment with each amino acid (m), (n), (o), and (p), respectively.

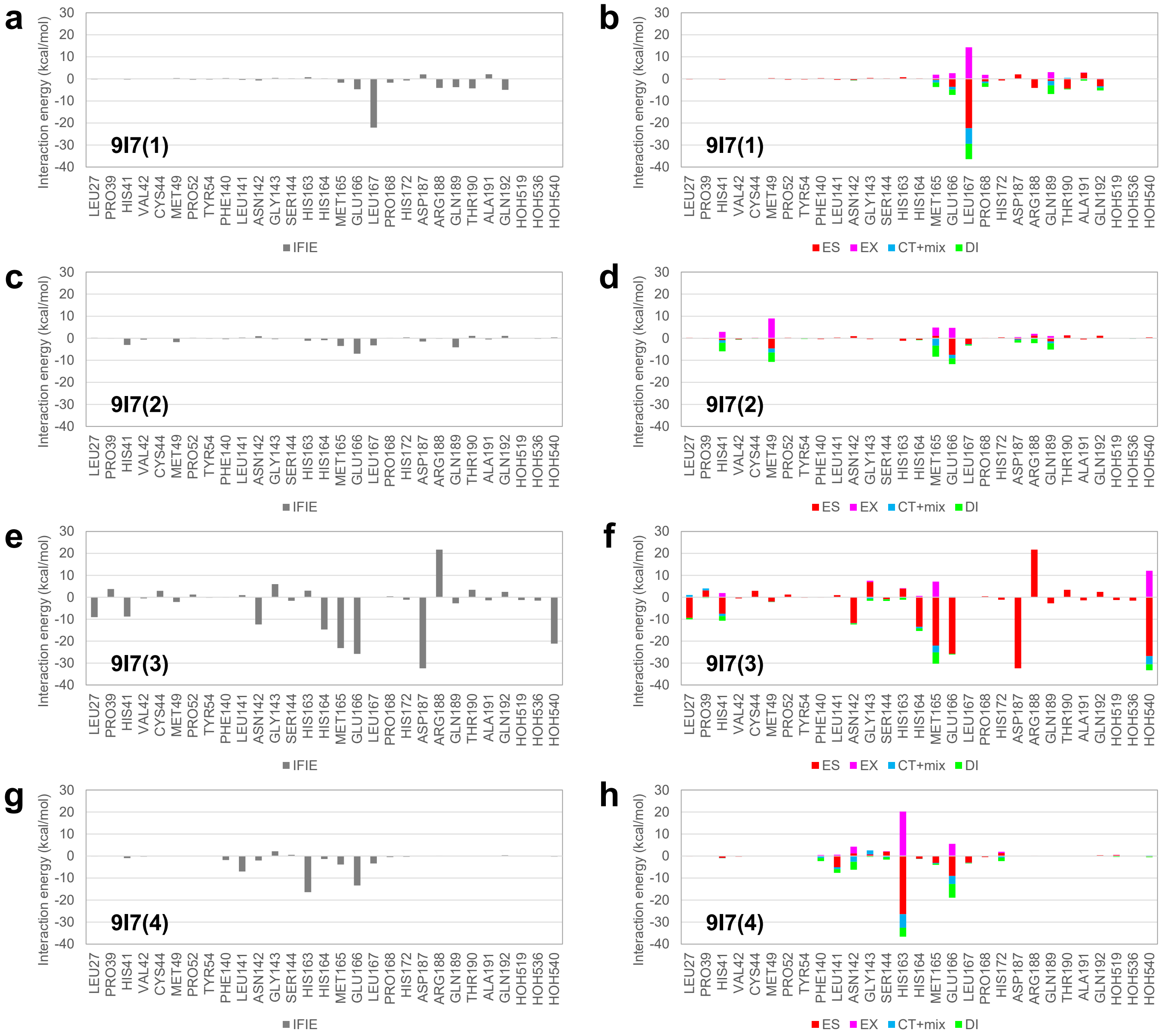

Figure S5. Interaction energy analysis between nirmatrelvir (9I7) and each amino acid residue of SARS-CoV-2 M^{pro} using ligand fragmentation. Interfragment interaction energy (IFIE) analyses of 9I7(1), 9I7(2), 9I7(3), and 9I7(4) are shown in (a), (c), (e), and (g), respectively, where fragments within 4.5 Å around the ligand were analyzed. Pair interaction energy decomposition analyses (PIEDA) of 9I7(1), 9I7(2), 9I7(3), and 9I7(4) are also shown in (b), (d), (f), and (h), respectively.

S3. Details of the accuracy verification of the fragmentation schema for four histidines coordinated with the Zn^{2+} ion

Table S2. Total time and total energies of FMO calculations, E_{total}, at MP2/6-31G and MP2/6-31G* levels for three fragmentation schemes, main chain, main/side chain (CB–CA), and main/side chain (CB–CG) fragmentations, using 48 cores (Intel(R) Xeon(R) CPU E5-2670 v3 @ 2.30GHz).

	Total time (s)	Total energy (hartree)		Diff. of total energy from main chain fragmentation data (hartree)	
Fragmentation	MP2/6-31G	HF/6-31G	MP2/6-31G	HF/6-31G	MP2/6-31G
Main chain	22867.9	−14273.134259	−14297.832157	-	-
Main/Side chain (CB–CA)	4152.3	−14272.256573	−14296.936548	0.877686	0.895609
Main/Side chain (CB–CG)	3476.3	−14272.792494	−14297.470763	0.341765	0.361394
	Total time (s)	Total energy (hartree)		Diff. of total energy from main chain fragmentation data (hartree)	
Fragmentation	MP2/6-31G*	HF/6-31G*	MP2/6-31G*	HF/6-31G*	MP2/6-31G*
Main chain	190394.6	−14278.153634	−14314.526500	-	-
Main/Side chain (CB–CA)	22117.6	−14277.213611	−14313.567646	0.940023	0.958854
Main/Side chain (CB–CG)	19379.9	−14277.784439	−14314.142674	0.369196	0.383826

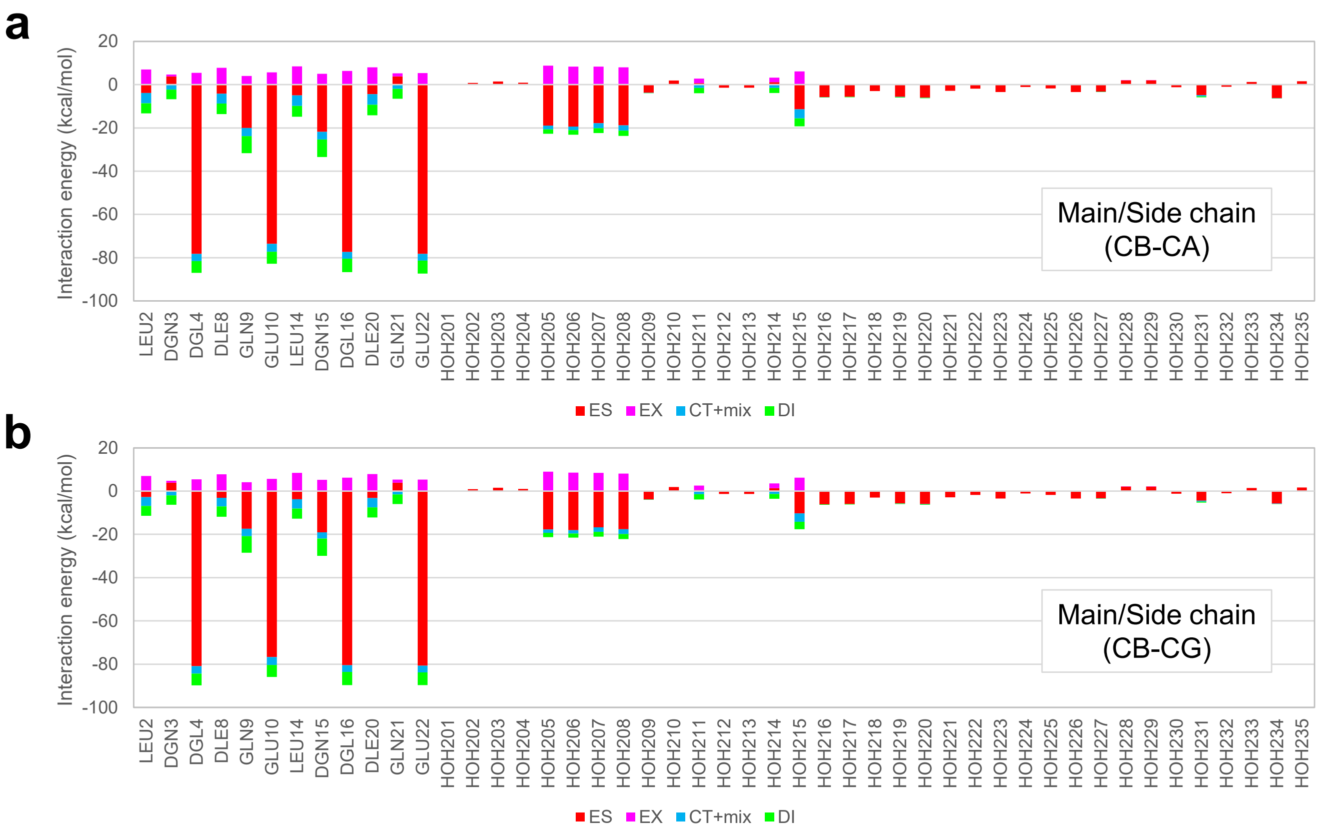

Figure S6. Interaction energy analysis of Zn^{2+} ion fragment including the four histidines for the cyclic peptide containing Zn^{2+} (PDB ID: 6UFA). Interaction energies with PIEDA for the main/side chain (CB–CA) fragmentation (FMODB ID: GYYM1) and the main/side chain (CB–CG) fragmentation (FMODB ID: 166QZ) are shown in (a) and (b), respectively. To compare the data with the main chain fragmentation data, the fragment units in the interaction energy analysis were adjusted to the main chain fragmentation scheme.

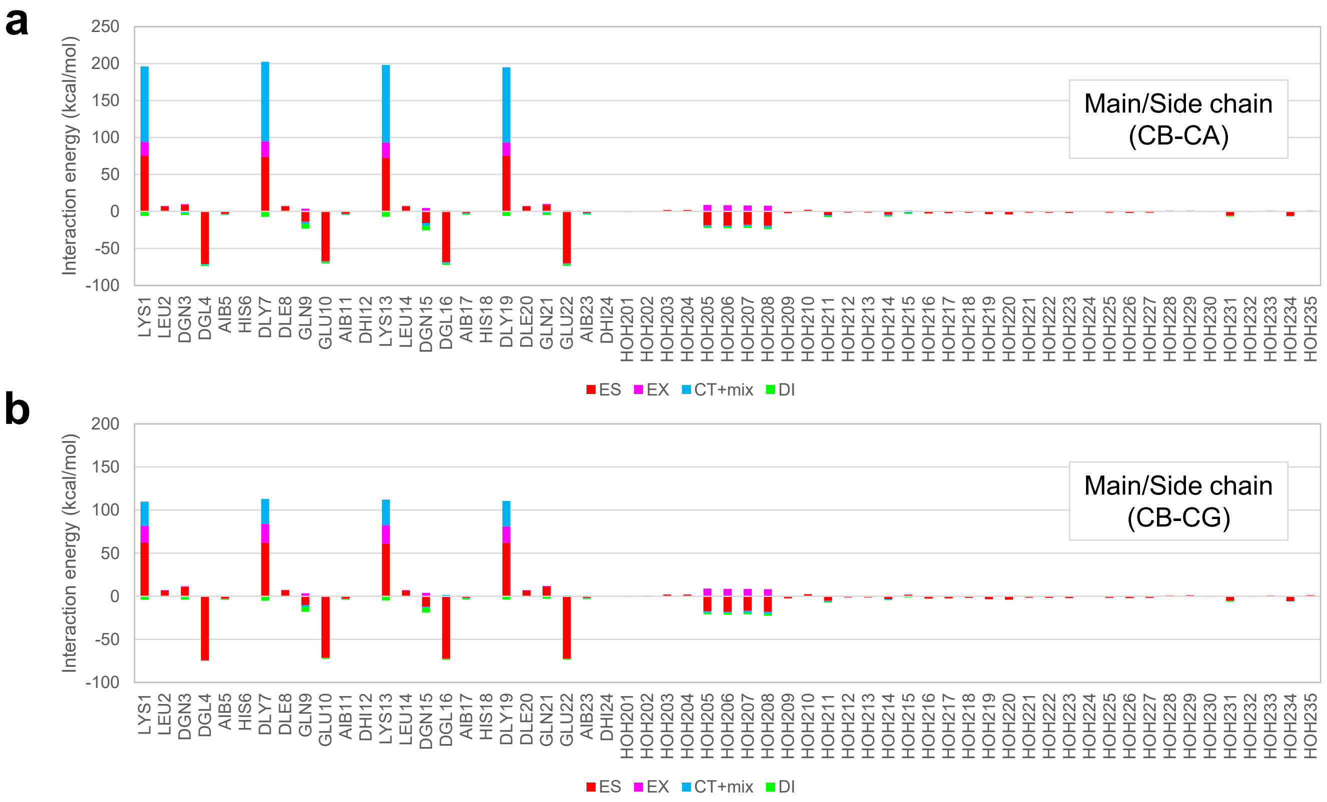

Figure S7. Interaction energy analysis of Zn^{2+} ion fragment including side chains of the four histidines for the cyclic peptide containing Zn^{2+} (PDB ID: 6UFA). Interaction energies with PIEDA for the main/side chain (CB–CA) fragmentation (FMODB ID: GYYM1) and the main/side chain (CB–CG) fragmentation (FMODB ID: 166QZ) are shown in (a) and (b), respectively. Interaction energy analysis was performed per fragment based on the main/side chain fragmentation scheme.

Table S3. Atomic charges of His6, His18, D-His12 (DHI12), D-His24 (DHI24), and Zn^{2+} ion using Mulliken, natural population analysis (NPA), and Merz–Kollman (MK) approaches at the FMO-HF/6-31G level.

			FMO-HF/6-31G								
			Mulliken			NPA			MK		
Atom #	Atom name	Residue	Main chain	Main /Side chain (CB-CA)	Main /Side chain (CB-CG)	Main chain	Main /Side chain (CB-CA)	Main /Side chain (CB-CG)	Main chain	Main /Side chain (CB-CA)	Main /Side chain (CB-CG)
87	N	HIS6	–0.928	–0.917	–0.923	–0.727	–0.741	–0.735	–0.914	–0.626	–1.644
88	CA	HIS6	0.012	0.033	–0.062	–0.086	–0.053	–0.090	–0.584	–0.131	0.654
89	C	HIS6	0.840	0.961	0.973	0.822	0.754	0.840	1.149	0.917	0.892
90	O	HIS6	–0.712	–0.741	–0.729	–0.790	–0.802	–0.779	–0.825	–0.766	–0.795
91	CB	HIS6	–0.280	–0.421	–0.229	–0.438	–0.180	–0.379	1.036	–0.268	–0.012
92	CG	HIS6	0.127	0.247	0.179	0.118	0.163	0.149	–0.342	0.589	0.510
93	ND1	HIS6	–0.961	–0.986	–0.983	–0.829	–0.843	–0.839	0.478	–0.798	–0.698
94	CD2	HIS6	0.183	0.130	0.126	–0.008	–0.069	–0.073	–0.233	–0.596	–0.693
95	CE1	HIS6	0.502	0.504	0.507	0.365	0.363	0.363	–0.006	–0.458	0.028
96	NE2	HIS6	–0.854	–0.850	–0.853	–0.498	–0.498	–0.499	–0.396	0.182	0.070
97	H	HIS6	0.461	0.475	0.467	0.484	0.490	0.487	0.430	0.300	0.569
98	HA	HIS6	0.260	0.278	0.247	0.250	0.251	0.244	0.284	0.075	0.052
99	HB2	HIS6	0.217	0.181	0.213	0.256	0.171	0.249	–0.101	0.118	0.063
100	HB3	HIS6	0.200	0.230	0.197	0.237	0.168	0.234	–0.359	0.003	–0.210
101	HD2	HIS6	0.245	0.236	0.237	0.220	0.217	0.217	0.270	0.245	0.325
102	HE1	HIS6	0.281	0.278	0.280	0.209	0.208	0.208	0.257	0.382	0.195
103	HE2	HIS6	0.424	0.417	0.424	0.436	0.434	0.435	0.543	0.393	0.435
293	N	HIS18	–0.949	–0.943	–0.942	–0.736	–0.751	–0.743	–0.738	–0.397	–1.174
294	CA	HIS18	0.026	0.063	–0.050	–0.079	–0.041	–0.092	0.001	–0.137	0.548
295	C	HIS18	0.839	0.954	0.969	0.830	0.763	0.850	0.901	0.937	0.987
296	O	HIS18	–0.720	–0.748	–0.738	–0.810	–0.821	–0.800	–0.803	–0.803	–0.863
297	CB	HIS18	–0.285	–0.404	–0.214	–0.436	–0.189	–0.379	0.492	–0.416	–0.366
298	CG	HIS18	0.140	0.243	0.173	0.136	0.175	0.167	–0.294	0.639	0.512
299	ND1	HIS18	–0.928	–0.950	–0.948	–0.802	–0.815	–0.811	0.142	–1.162	–0.708
300	CD2	HIS18	0.180	0.132	0.126	–0.010	–0.066	–0.069	–0.083	–0.418	–0.642
301	CE1	HIS18	0.488	0.488	0.489	0.345	0.346	0.346	0.235	0.136	0.006
302	NE2	HIS18	–0.876	–0.874	–0.877	–0.518	–0.518	–0.519	–0.506	–0.255	–0.075
303	H	HIS18	0.464	0.482	0.471	0.479	0.488	0.483	0.365	0.222	0.434
304	HA	HIS18	0.245	0.249	0.227	0.238	0.237	0.234	0.055	0.120	0.007
305	HB2	HIS18	0.269	0.228	0.261	0.278	0.197	0.270	0.045	0.176	0.233
306	HB3	HIS18	0.185	0.209	0.182	0.231	0.160	0.228	–0.180	0.059	–0.019
307	HD2	HIS18	0.265	0.255	0.256	0.224	0.221	0.222	0.295	0.298	0.421
308	HE1	HIS18	0.258	0.255	0.257	0.204	0.202	0.202	0.146	0.179	0.252
309	HE2	HIS18	0.419	0.412	0.418	0.428	0.425	0.426	0.536	0.482	0.467

190	N	DHI12	–0.931	–0.920	–0.923	–0.732	–0.747	–0.739	–0.660	–0.501	–1.432
191	CA	DHI12	0.016	0.044	–0.060	–0.086	–0.052	–0.091	–0.462	–0.171	0.497
192	C	DHI12	0.838	0.954	0.967	0.821	0.757	0.842	1.108	0.928	0.989
193	O	DHI12	–0.709	–0.738	–0.726	–0.787	–0.799	–0.776	–0.798	–0.762	–0.820
194	CB	DHI12	–0.284	–0.425	–0.213	–0.439	–0.186	–0.387	–0.038	–0.551	–0.448
195	CG	DHI12	0.150	0.266	0.184	0.143	0.180	0.173	–0.060	0.555	0.479
196	ND1	DHI12	–0.932	–0.952	–0.951	–0.802	–0.815	–0.812	0.471	–0.826	–0.553
197	CD2	DHI12	0.180	0.130	0.128	–0.024	–0.080	–0.084	–0.343	–0.527	–0.597
198	CE1	DHI12	0.498	0.501	0.501	0.346	0.353	0.352	–0.005	–0.077	0.092
199	NE2	DHI12	–0.861	–0.859	–0.862	–0.513	–0.513	–0.515	–0.303	–0.060	–0.081
200	H	DHI12	0.452	0.467	0.459	0.476	0.483	0.480	0.376	0.262	0.536
201	HA	DHI12	0.258	0.273	0.243	0.250	0.251	0.244	0.254	0.132	0.163
202	HB2	DHI12	0.242	0.206	0.237	0.266	0.182	0.259	0.205	0.265	0.316
203	HB3	DHI12	0.198	0.227	0.190	0.237	0.166	0.234	0.022	0.130	0.009
204	HD2	DHI12	0.232	0.223	0.222	0.215	0.212	0.212	0.311	0.285	0.329
205	HE1	DHI12	0.250	0.246	0.249	0.206	0.205	0.204	0.152	0.180	0.161
206	HE2	DHI12	0.419	0.412	0.417	0.432	0.429	0.430	0.486	0.408	0.447
396	N	DHI24	–0.946	–0.942	–0.942	–0.737	–0.753	–0.745	–0.682	–0.361	–1.161
397	CA	DHI24	0.027	0.055	–0.049	–0.079	–0.046	–0.092	–0.323	–0.174	0.441
398	C	DHI24	0.839	0.957	0.973	0.832	0.766	0.851	1.120	1.033	1.028
399	O	DHI24	–0.723	–0.752	–0.742	–0.810	–0.821	–0.800	–0.919	–0.902	–0.926
400	CB	DHI24	–0.286	–0.408	–0.237	–0.436	–0.183	–0.374	0.550	–0.551	–0.419
401	CG	DHI24	0.130	0.244	0.179	0.114	0.156	0.142	–0.236	0.629	0.569
402	ND1	DHI24	–0.958	–0.983	–0.980	–0.830	–0.844	–0.841	0.437	–0.979	–0.629
403	CD2	DHI24	0.169	0.116	0.113	–0.006	–0.063	–0.067	–0.104	–0.359	–0.629
404	CE1	DHI24	0.487	0.487	0.490	0.358	0.355	0.355	0.199	0.020	0.208
405	NE2	DHI24	–0.845	–0.842	–0.845	–0.501	–0.501	–0.502	–0.677	–0.271	–0.208
406	H	DHI24	0.466	0.483	0.473	0.483	0.491	0.486	0.322	0.223	0.468
407	HA	DHI24	0.244	0.250	0.227	0.239	0.238	0.234	0.163	0.102	0.081
408	HB2	DHI24	0.243	0.204	0.240	0.265	0.183	0.258	–0.004	0.230	0.186
409	HB3	DHI24	0.194	0.220	0.193	0.234	0.164	0.230	–0.204	0.106	0.028
410	HD2	DHI24	0.264	0.259	0.261	0.226	0.224	0.224	0.244	0.210	0.351
411	HE1	DHI24	0.282	0.279	0.281	0.211	0.210	0.210	0.206	0.280	0.157
412	HE2	DHI24	0.421	0.415	0.422	0.443	0.441	0.442	0.621	0.498	0.509
413	ZN	ZN101	1.568	1.581	1.555	1.743	1.728	1.723	–1.121	2.674	0.806

Table S4. Atomic charges of His6, His18, D-His12 (DHI12), D-His24 (DHI24), and Zn^{2+} ion using Mulliken, natural population analysis (NPA), and Merz–Kollman (MK) approaches at the FMO-HF/6-31G* level.

			FMO-HF/6-31G*								
			Mulliken			NPA			MK		
Atom #	Atom name	Residue	Main chain	Main /Side chain (CB-CA)	Main /Side chain (CB-CG)	Main chain	Main /Side chain (CB-CA)	Main /Side chain (CB-CG)	Main chain	Main /Side chain (CB-CA)	Main /Side chain (CB-CG)
87	N	HIS6	–0.851	–0.845	–0.847	–0.729	–0.741	–0.737	–0.880	–0.655	–1.492
88	CA	HIS6	–0.025	–0.002	–0.090	–0.074	–0.048	–0.083	–0.437	–0.166	0.534
89	C	HIS6	0.810	0.914	0.925	0.867	0.803	0.890	0.869	0.818	0.780
90	O	HIS6	–0.701	–0.731	–0.719	–0.810	–0.821	–0.800	–0.727	–0.700	–0.716
91	CB	HIS6	–0.345	–0.490	–0.282	–0.417	–0.167	–0.363	1.020	–0.071	0.034
92	CG	HIS6	0.179	0.287	0.211	0.128	0.167	0.158	–0.369	0.401	0.421
93	ND1	HIS6	–0.878	–0.902	–0.899	–0.840	–0.854	–0.851	0.420	–0.734	–0.523
94	CD2	HIS6	0.023	–0.029	–0.029	0.003	–0.052	–0.057	–0.214	–0.516	–0.666
95	CE1	HIS6	0.371	0.372	0.376	0.401	0.401	0.401	–0.141	–0.411	–0.070
96	NE2	HIS6	–0.693	–0.687	–0.691	–0.503	–0.502	–0.503	–0.356	0.099	0.033
97	H	HIS6	0.464	0.478	0.469	0.471	0.478	0.474	0.428	0.338	0.551
98	HA	HIS6	0.260	0.275	0.252	0.233	0.231	0.227	0.260	0.107	0.080
99	HB2	HIS6	0.239	0.223	0.237	0.247	0.167	0.239	–0.104	0.092	0.050
100	HB3	HIS6	0.208	0.251	0.202	0.221	0.157	0.217	–0.378	–0.081	–0.194
101	HD2	HIS6	0.243	0.234	0.235	0.208	0.204	0.205	0.257	0.239	0.326
102	HE1	HIS6	0.268	0.265	0.266	0.192	0.191	0.191	0.307	0.360	0.223
103	HE2	HIS6	0.460	0.453	0.459	0.425	0.422	0.423	0.569	0.440	0.480
293	N	HIS18	–0.874	–0.872	–0.868	–0.737	–0.751	–0.745	–0.624	–0.382	–0.933
294	CA	HIS18	–0.018	0.019	–0.086	–0.068	–0.043	–0.086	0.106	–0.109	0.452
295	C	HIS18	0.804	0.903	0.916	0.873	0.810	0.896	0.738	0.902	0.920
296	O	HIS18	–0.704	–0.733	–0.724	–0.828	–0.838	–0.820	–0.744	–0.766	–0.809
297	CB	HIS18	–0.350	–0.476	–0.272	–0.414	–0.170	–0.364	0.341	–0.224	–0.343
298	CG	HIS18	0.192	0.284	0.202	0.144	0.178	0.174	–0.299	0.410	0.439
299	ND1	HIS18	–0.850	–0.871	–0.869	–0.815	–0.828	–0.825	0.194	–1.011	–0.585
300	CD2	HIS18	0.021	–0.025	–0.027	0.001	–0.049	–0.054	–0.077	–0.384	–0.654
301	CE1	HIS18	0.362	0.363	0.366	0.385	0.387	0.388	0.084	0.108	–0.048
302	NE2	HIS18	–0.712	–0.707	–0.712	–0.521	–0.519	–0.521	–0.581	–0.345	–0.170
303	H	HIS18	0.470	0.487	0.476	0.468	0.476	0.471	0.341	0.230	0.373
304	HA	HIS18	0.252	0.254	0.239	0.224	0.220	0.219	0.031	0.097	0.014
305	HB2	HIS18	0.277	0.253	0.273	0.263	0.187	0.255	0.103	0.169	0.244
306	HB3	HIS18	0.197	0.236	0.194	0.216	0.150	0.213	–0.140	–0.018	0.003
307	HD2	HIS18	0.258	0.248	0.250	0.211	0.208	0.209	0.310	0.334	0.461
308	HE1	HIS18	0.247	0.244	0.246	0.188	0.186	0.186	0.184	0.171	0.266
309	HE2	HIS18	0.454	0.446	0.452	0.416	0.413	0.414	0.661	0.588	0.582

190	N	DHI12	–0.858	–0.851	–0.851	–0.734	–0.748	–0.741	–0.538	–0.492	–1.208
191	CA	DHI12	–0.021	0.007	–0.087	–0.075	–0.050	–0.085	–0.390	–0.166	0.407
192	C	DHI12	0.809	0.905	0.917	0.867	0.805	0.891	0.913	0.890	0.901
193	O	DHI12	–0.697	–0.726	–0.714	–0.806	–0.817	–0.796	–0.723	–0.714	–0.751
194	CB	DHI12	–0.349	–0.495	–0.269	–0.416	–0.169	–0.372	0.062	–0.400	–0.403
195	CG	DHI12	0.205	0.306	0.216	0.152	0.183	0.181	–0.187	0.376	0.438
196	ND1	DHI12	–0.852	–0.872	–0.870	–0.816	–0.829	–0.826	0.435	–0.729	–0.549
197	CD2	DHI12	0.018	–0.030	–0.029	–0.012	–0.062	–0.068	–0.265	–0.471	–0.578
198	CE1	DHI12	0.370	0.373	0.375	0.385	0.394	0.393	–0.090	–0.129	0.060
199	NE2	DHI12	–0.695	–0.690	–0.695	–0.516	–0.515	–0.517	–0.351	–0.087	–0.109
200	H	DHI12	0.458	0.473	0.463	0.466	0.473	0.469	0.359	0.296	0.500
201	HA	DHI12	0.259	0.273	0.250	0.234	0.232	0.228	0.233	0.125	0.154
202	HB2	DHI12	0.255	0.239	0.252	0.253	0.175	0.246	0.204	0.255	0.298
203	HB3	DHI12	0.205	0.248	0.197	0.221	0.156	0.217	–0.040	0.049	0.018
204	HD2	DHI12	0.232	0.223	0.223	0.203	0.200	0.201	0.293	0.291	0.340
205	HE1	DHI12	0.238	0.234	0.237	0.189	0.188	0.188	0.171	0.192	0.175
206	HE2	DHI12	0.450	0.442	0.448	0.420	0.417	0.418	0.570	0.464	0.505
396	N	DHI24	–0.870	–0.869	–0.866	–0.739	–0.753	–0.747	–0.604	–0.381	–0.941
397	CA	DHI24	–0.015	0.011	–0.085	–0.069	–0.044	–0.086	–0.216	–0.173	0.349
398	C	DHI24	0.806	0.909	0.924	0.877	0.813	0.899	0.932	0.970	0.933
399	O	DHI24	–0.707	–0.736	–0.727	–0.828	–0.839	–0.820	–0.850	–0.853	–0.862
400	CB	DHI24	–0.352	–0.476	–0.290	–0.414	–0.167	–0.357	0.433	–0.412	–0.378
401	CG	DHI24	0.176	0.277	0.202	0.122	0.159	0.150	–0.079	0.570	0.567
402	ND1	DHI24	–0.874	–0.897	–0.894	–0.841	–0.854	–0.851	0.173	–1.018	–0.681
403	CD2	DHI24	0.021	–0.030	–0.030	0.007	–0.044	–0.049	–0.105	–0.413	–0.650
404	CE1	DHI24	0.358	0.359	0.363	0.395	0.393	0.394	0.255	0.170	0.328
405	NE2	DHI24	–0.688	–0.682	–0.686	–0.506	–0.505	–0.505	–0.773	–0.363	–0.320
406	H	DHI24	0.471	0.486	0.476	0.471	0.478	0.474	0.300	0.234	0.397
407	HA	DHI24	0.249	0.252	0.237	0.224	0.221	0.219	0.144	0.114	0.096
408	HB2	DHI24	0.257	0.235	0.257	0.252	0.175	0.244	0.037	0.225	0.196
409	HB3	DHI24	0.207	0.246	0.204	0.219	0.154	0.215	–0.188	0.048	0.031
410	HD2	DHI24	0.258	0.251	0.253	0.212	0.209	0.210	0.235	0.267	0.388
411	HE1	DHI24	0.271	0.268	0.269	0.194	0.193	0.193	0.212	0.221	0.135
412	HE2	DHI24	0.454	0.448	0.454	0.432	0.430	0.431	0.679	0.537	0.556
413	ZN	ZN101	1.517	1.529	1.513	1.737	1.719	1.717	–0.638	2.781	0.748

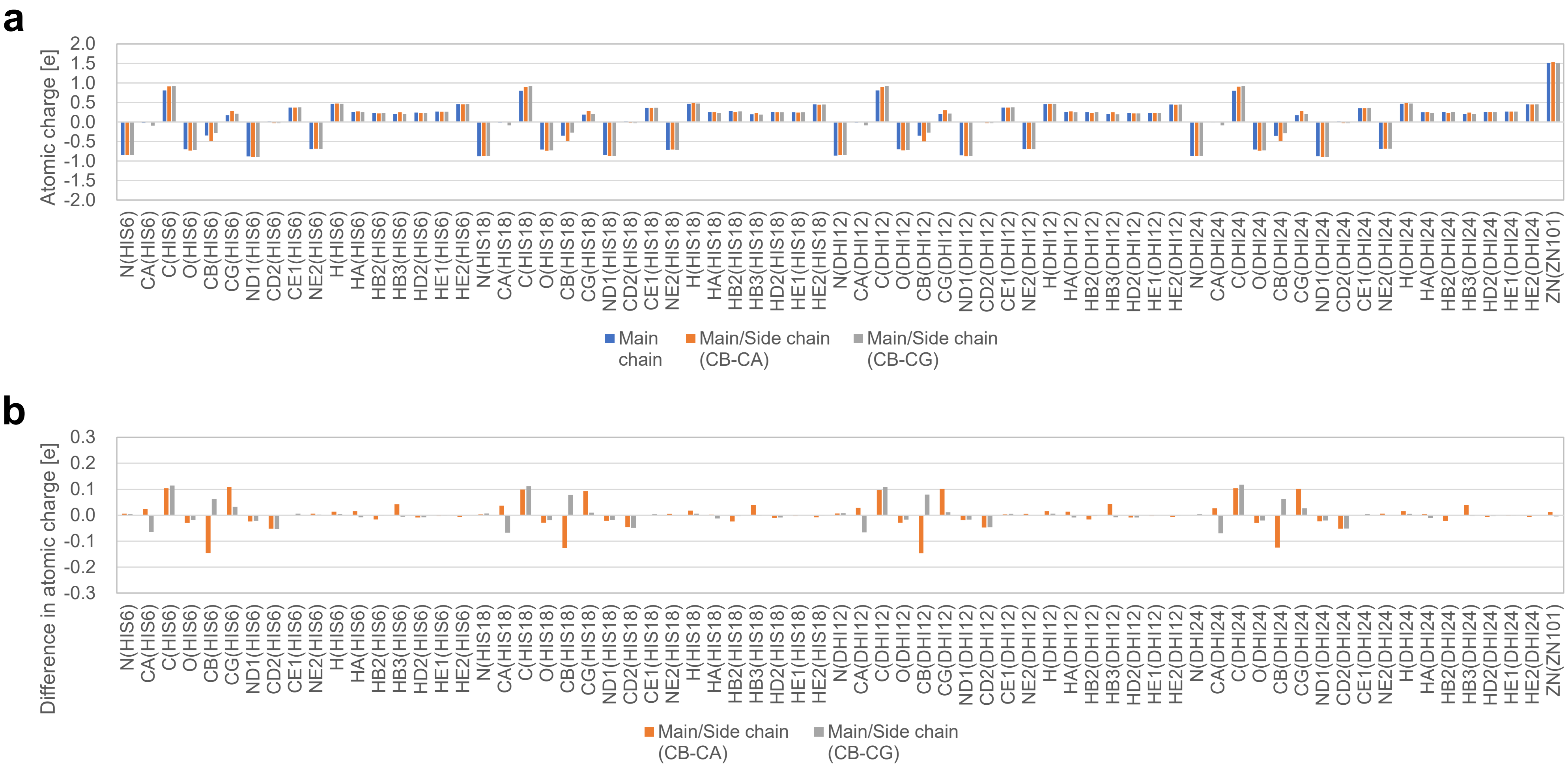

Figure S8. Mulliken population analysis for each fragmentation scheme at the HF/6-31G* level. The net atomic charges for the three fragmentation schemes are shown in (a); the differences in the atomic charge between the main chain fragmentation and the main/side chain fragmentation data are shown in (b).

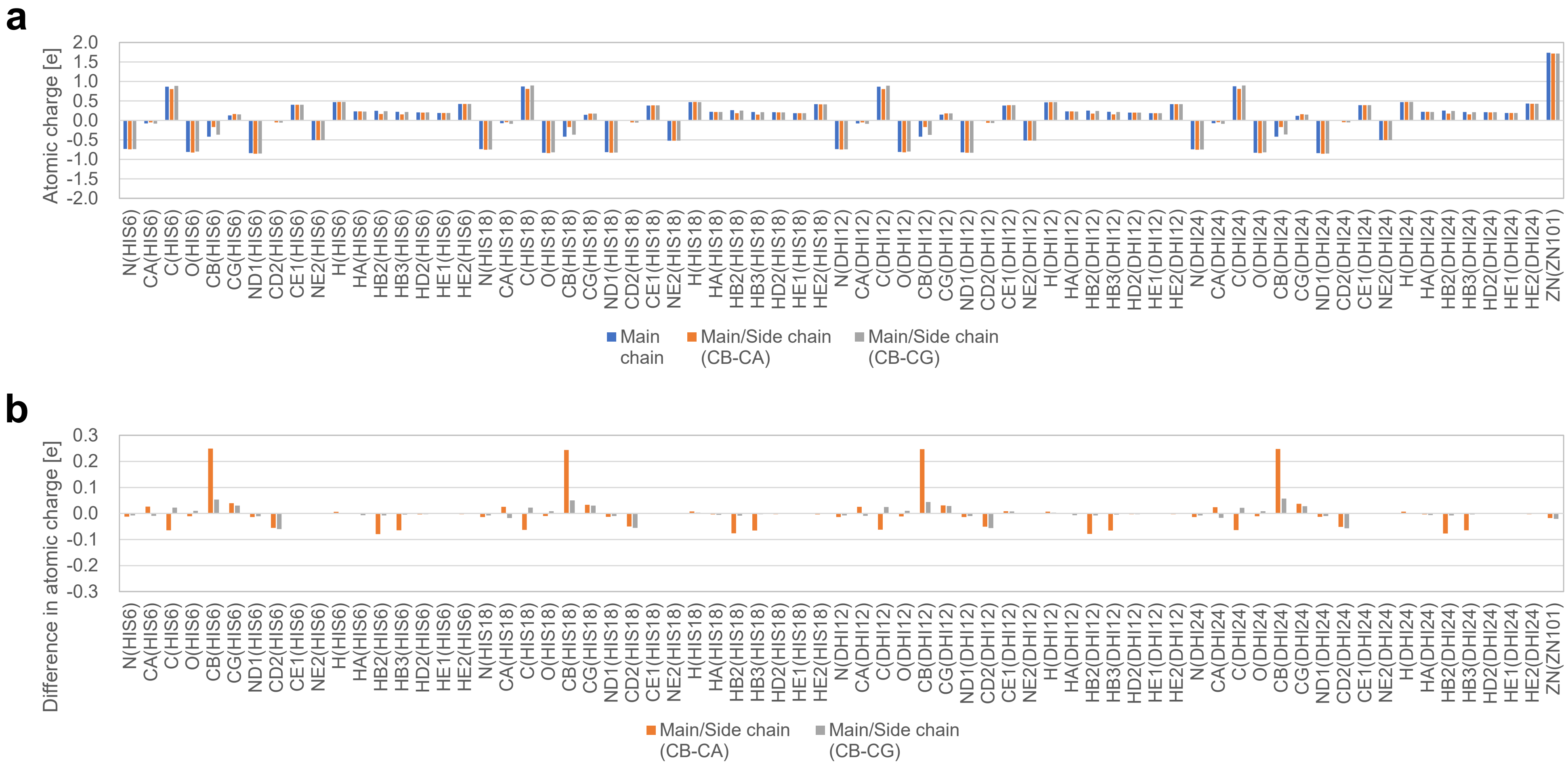

Figure S9. Natural population analysis for each fragmentation scheme at the HF/6-31G* level. The net atomic charges for the three fragmentation schemes are shown in (a); the differences in the atomic charge between the main chain fragmentation and the main/side chain fragmentation data are shown in (b).

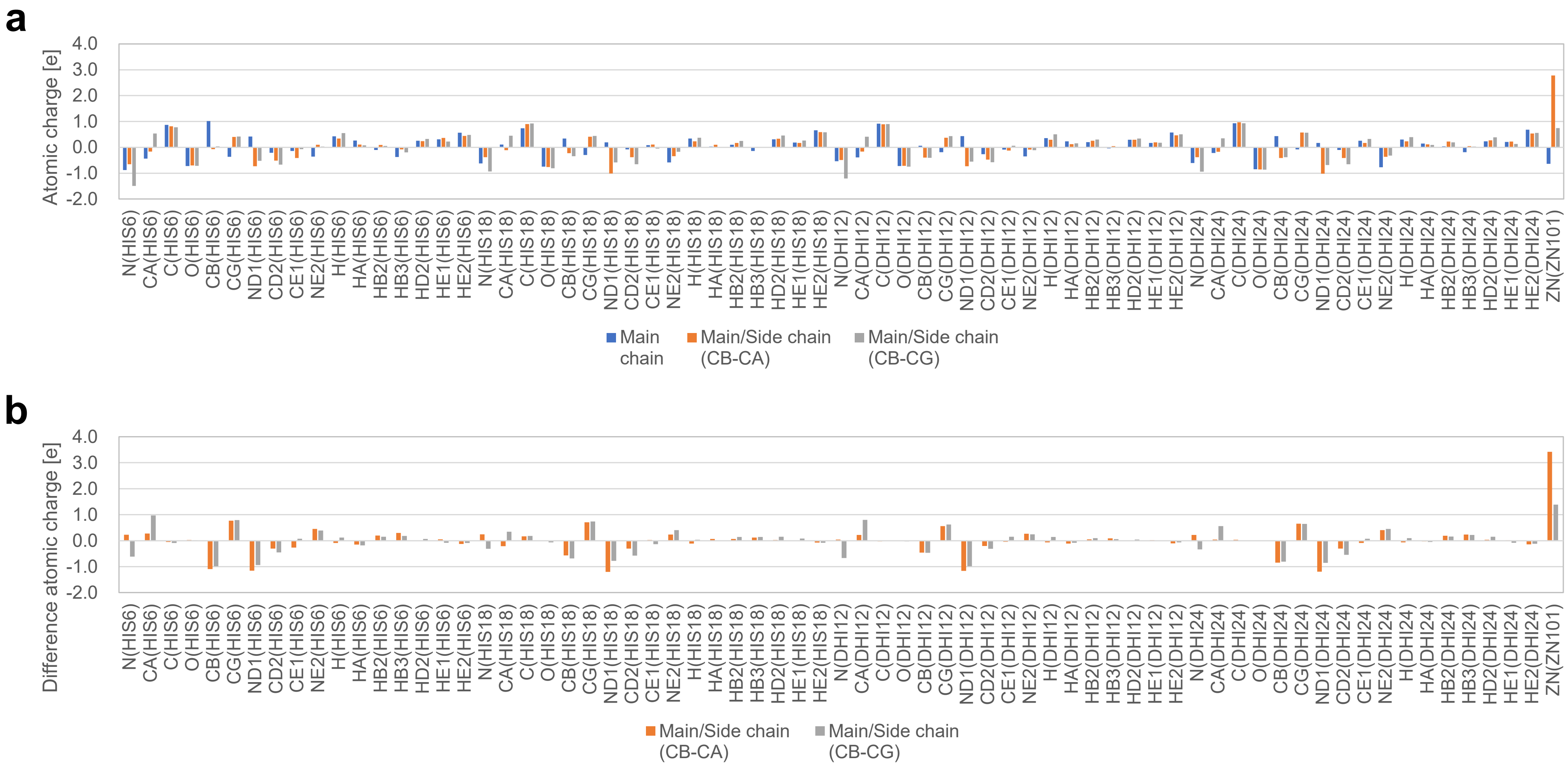

Figure S10. Merz–Kollman charge analysis for each fragmentation scheme at the HF/6-31G* level. The net atomic charges for the three fragmentation schemes are shown in (a); the differences in the atomic charge between the main chain fragmentation and the main/side chain fragmentation data are shown in (b).

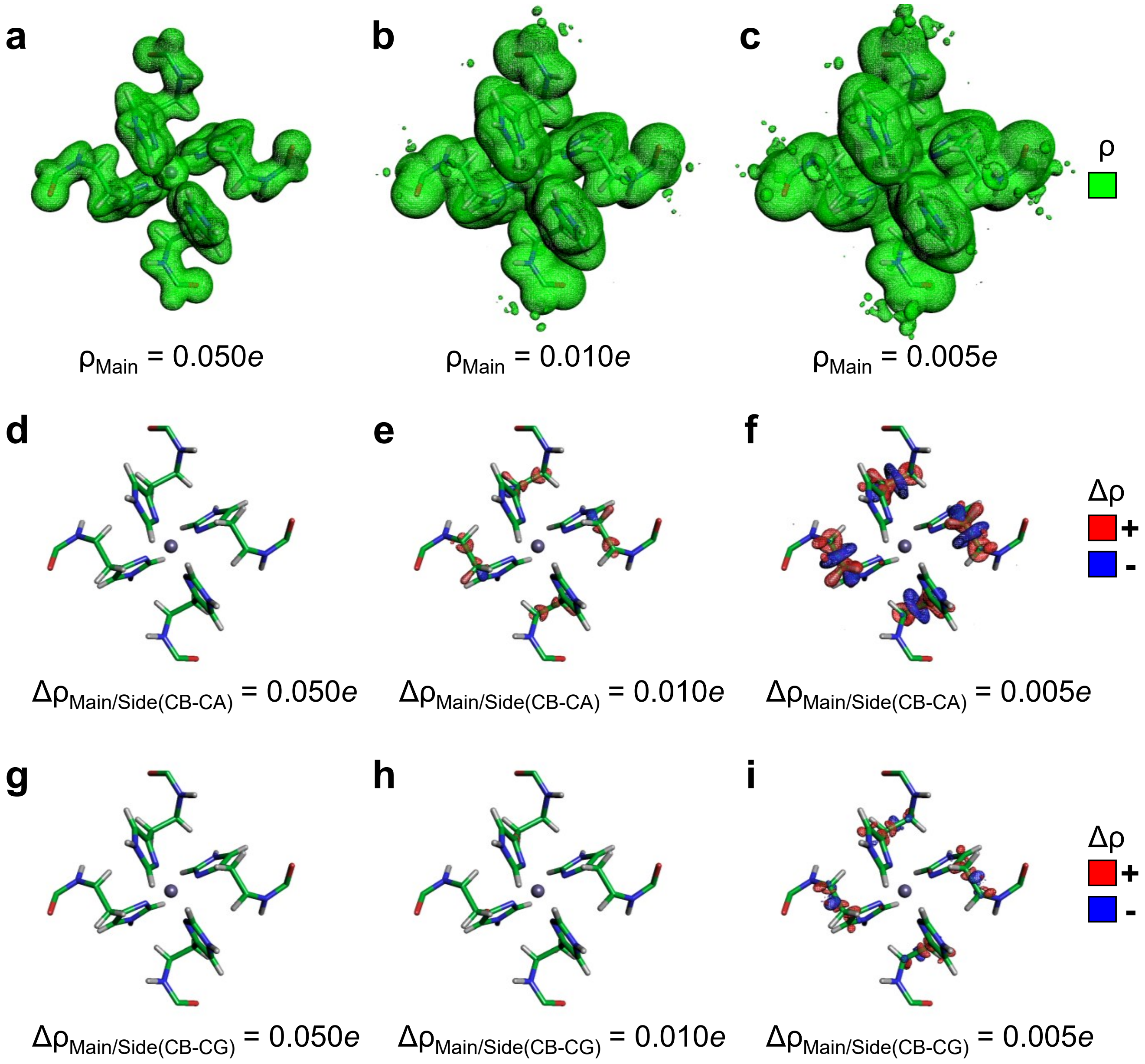

Figure S11. FMO-based electron density analysis of a Zn^{2+} ion and its coordinated His. For the main-chain fragmentation, the electron densities (ρ) at 0.050e, 0.010e, and 0.005e are shown in (a), (b), and (c), respectively. For the main/side chain fragmentation (CB–CA), the difference electron densities ($\Delta\rho$) from the main chain data at 0.050e, 0.010e, and 0.005e are shown in (d), (e), and (f), respectively. For the main/side-chain fragmentation (CB–CG), the difference electron densities ($\Delta\rho$) from the main-chain data at 0.050e, 0.010e, and 0.005e are shown in (g), (h), and (i), respectively. The electron density was drawn using BioStation Viewer[14,26,32].

References

(1) Kitaura, K.; Ikeo, E.; Asada, T.; Nakano, T.; Uebayasi, M. Fragment Molecular Orbital Method: An Approximate Computational Method for Large Molecules. *Chemical Physics Letters* **1999**, *313* (3–4), 701–706. https://doi.org/10.1016/S0009-2614(99)00874-X.

(2) Mochizuki, Y.; Koikegami, S.; Nakano, T.; Amari, S.; Kitaura, K. Large Scale MP2 Calculations with Fragment Molecular Orbital Scheme. *Chemical Physics Letters* **2004**, *396* (4–6), 473–479. https://doi.org/10.1016/j.cplett.2004.08.082.

(3) Fedorov, D. G.; Kitaura, K. Pair Interaction Energy Decomposition Analysis. *J. Comput. Chem.* **2007**, *28* (1), 222–237. https://doi.org/10.1002/jcc.20496.

(4) Tsukamoto T.; Kato K.; Kato A.; Nakano T.; Mochizuki Y.; Fukuzawa K. Implementation of Pair Interaction Energy DecompositionAnalysis and Its Applications to Protein-Ligand Systems. *J. Comput. Chem. Jpn.* **2015**, *14* (1), 1–9. https://doi.org/10.2477/jccj.2014-0039.

(5) Okiyama, Y.; Watanabe, C.; Fukuzawa, K.; Mochizuki, Y.; Nakano, T.; Tanaka, S. Fragment Molecular Orbital Calculations with Implicit Solvent Based on the Poisson–Boltzmann Equation: II. Protein and Its Ligand-Binding System Studies. *J. Phys. Chem. B* **2019**, *123* (5), 957–973. https://doi.org/10.1021/acs.jpcb.8b09326.

(6) Watanabe, C.; Okiyama, Y.; Tanaka, S.; Fukuzawa, K.; Honma, T. Molecular Recognition of SARS-CoV-2 Spike Glycoprotein: Quantum Chemical Hot Spot and Epitope Analyses. **2020**. https://doi.org/10.26434/chemrxiv.13200176.v3.

(7) Nakano, T.; Kaminuma, T.; Sato, T.; Akiyama, Y.; Uebayasi, M.; Kitaura, K. Fragment Molecular Orbital Method: Application to Polypeptides. *Chemical Physics Letters* **2000**, *318* (6), 614–618. https://doi.org/10.1016/S0009-2614(00)00070-1.

(8) Kitaura, K. FU. https://cbi-society.org/home/documents/FU.html (accessed 2024-07-21).

(9) Fedorov, D. G.; Kitaura, K. Modeling and Visualization for the Fragment Molecular Orbital Method with the Graphical User Interface FU, and Analyses of Protein–Ligand Binding. In *Fragmentation*; John Wiley & Sons, Ltd, 2017; pp 119–139. https://doi.org/10.1002/9781119129271.ch3.

(10) Suenaga, M. Facio. https://zzzfelis.sakura.ne.jp/ (accessed 2024-07-21).

(11) Suenaga, M. Facio: New Computational Chemistry Environment for PC GAMESS. *Journal of Computer Chemistry, Japan* **2005**, *4* (1), 25–32. https://doi.org/10.2477/jccj.4.25.

(12) Suenaga, M. Development of GUI for GAMESS / FMO Calculation. *Journal of Computer Chemistry, Japan* **2008**, *7* (1), 33–54. https://doi.org/10.2477/jccj.H1920.

(13) Nakamura, S. MOE-FMOutil, 2011. https://svl.chemcomp.com/#MOE_FMOutil (accessed 2024-07-21).

(14) Kato, A. *BioStation Viewer*. https://fmodd.jp/biostationviewer-dl/ (accessed 2024-07-21).

(15) Moriwaki, H.; Kawashima, Y.; Watanabe, C.; Kamisaka, K. FMOe, 2018. https://github.com/drugdesign/FMOe (accessed 2024-07-21).

(16) Kimura, Y. View PIEDA, 2022. https://svl.chemcomp.com/#ViewPieda (accessed 2024-07-21).

(17) PAICS View. http://www.paics.net/paics_view_e.html (accessed 2024-07-21).

(18) Tsuji, M. Virtual Screening and Quantum Chemistry Analysis for SARS-CoV-2 RNA-Dependent RNA Polymerase Using the ChEMBL Database: Reproduction of the Remdesivir-RTP and Favipiravir-RTP Binding Modes Obtained from Cryo-EM Experiments with High Binding Affinity. *IJMS* **2022**, *23* (19), 11009. https://doi.org/10.3390/ijms231911009.

(19) Tokiwa, T.; Nakano, S.; Yamamoto, Y.; Ishikawa, T.; Ito, S.; Sladek, V.; Fukuzawa, K.; Mochizuki, Y.; Tokiwa, H.; Misaizu, F.; Shigeta, Y. Development of an Analysis Toolkit, AnalysisFMO, to Visualize Interaction Energies Generated by Fragment Molecular Orbital Calculations. *J. Chem. Inf. Model.* **2019**, *59* (1), 25–30. https://doi.org/10.1021/acs.jcim.8b00649.

(20) *Gordon Group/GAMESS Homepage*. https://www.msg.chem.iastate.edu/gamess/index.html (accessed 2024-07-21).

(21) Barca, G. M. J.; Bertoni, C.; Carrington, L.; Datta, D.; De Silva, N.; Deustua, J. E.; Fedorov, D. G.; Gour, J. R.; Gunina, A. O.; Guidez, E.; Harville, T.; Irle, S.; Ivanic, J.; Kowalski, K.; Leang, S. S.; Li, H.; Li, W.; Lutz, J. J.; Magoulas, I.; Mato, J.; Mironov, V.; Nakata, H.; Pham, B. Q.; Piecuch, P.; Poole, D.; Pruitt, S. R.; Rendell, A. P.; Roskop, L. B.; Ruedenberg, K.; Sattasathuchana, T.; Schmidt, M. W.; Shen, J.; Slipchenko, L.; Sosonkina, M.; Sundriyal, V.; Tiwari, A.; Galvez Vallejo, J. L.; Westheimer, B.; Włoch, M.; Xu, P.; Zahariev, F.; Gordon, M. S. Recent Developments in the General Atomic and Molecular Electronic Structure System. *J. Chem. Phys.* **2020**, *152* (15), 154102. https://doi.org/10.1063/5.0005188.

(22) Fedorov, D. G. Recent Development of the Fragment Molecular Orbital Method in GAMESS. In *Recent Advances of the Fragment Molecular Orbital Method: Enhanced Performance and Applicability*; Mochizuki, Y., Tanaka, S., Fukuzawa, K., Eds.; Springer: Singapore, 2021; pp 31–51. https://doi.org/10.1007/978-981-15-9235-5_3.

(23) Fedorov, D. G. *Complete Guide To The Fragment Molecular Orbital Method In Gamess: From One Atom To A Million, At Your Service*; World Scientific, 2023.

(24) Nakano, T.; Mochizuki, Y.; Fukuzawa, K.; Amari, S.; Tanaka, S. CHAPTER 2 - Developments and Applications of ABINIT-MP Software Based on the Fragment Molecular Orbital Method. In *Modern Methods for Theoretical Physical Chemistry of Biopolymers*; Starikov, E. B., Lewis, J. P., Tanaka, S., Eds.; Elsevier Science: Amsterdam, 2006; pp 39–52. https://doi.org/10.1016/B978-044452220-7/50066-6.

(25) Tanaka, S.; Mochizuki, Y.; Komeiji, Y.; Okiyama, Y.; Fukuzawa, K. Electron-Correlated Fragment-Molecular-Orbital Calculations for Biomolecular and Nano Systems. *Phys. Chem. Chem. Phys.* **2014**, *16* (22), 10310–10344. https://doi.org/10.1039/C4CP00316K.

(26) Mochizuki, Y.; Nakano, T.; Sakakura, K.; Okiyama, Y.; Watanabe, H.; Kato, K.; Akinaga, Y.; Sato, S.; Yamamoto, J.; Yamashita, K.; Murase, T.; Ishikawa, T.; Komeiji, Y.; Kato, Y.; Watanabe, N.; Tsukamoto, T.; Mori, H.; Okuwaki, K.; Tanaka, S.; Kato, A.; Watanabe, C.; Fukuzawa, K. The ABINIT-MP Program. *Recent Advances of the Fragment Molecular Orbital Method: Enhanced Performance and Applicability*, 2021, 53–67. https://doi.org/10.1007/978-981-15-9235-5_4.

(27) Takeshi, I. *PAICS - parallelized ab initio calculation system based on FMO*. http://www.paics.net/index_e.html (accessed 2024-07-18).
(28) Ishikawa, T. PAICS: Development of an Open-Source Software of Fragment Molecular Orbital Method for Biomolecule. In *Recent Advances of the Fragment Molecular Orbital Method: Enhanced Performance and Applicability*; Mochizuki, Y., Tanaka, S., Fukuzawa, K., Eds.; Springer: Singapore, 2021; pp 69–76. https://doi.org/10.1007/978-981-15-9235-5_5.
(29) Owen, D. R.; Allerton, C. M. N.; Anderson, A. S.; Aschenbrenner, L.; Avery, M.; Berritt, S.; Boras, B.; Cardin, R. D.; Carlo, A.; Coffman, K. J.; Dantonio, A.; Di, L.; Eng, H.; Ferre, R.; Gajiwala, K. S.; Gibson, S. A.; Greasley, S. E.; Hurst, B. L.; Kadar, E. P.; Kalgutkar, A. S.; Lee, J. C.; Lee, J.; Liu, W.; Mason, S. W.; Noell, S.; Novak, J. J.; Obach, R. S.; Ogilvie, K.; Patel, N. C.; Pettersson, M.; Rai, D. K.; Reese, M. R.; Sammons, M. F.; Sathish, J. G.; Singh, R. S. P.; Steppan, C. M.; Stewart, A. E.; Tuttle, J. B.; Updyke, L.; Verhoest, P. R.; Wei, L.; Yang, Q.; Zhu, Y. An Oral SARS-CoV-2 Mpro Inhibitor Clinical Candidate for the Treatment of COVID-19. *Science* **2021**, *374* (6575), 1586–1593. https://doi.org/10.1126/science.abl47.
(30) Kneller, D. W.; Li, H.; Phillips, G.; Weiss, K. L.; Zhang, Q.; Arnould, M. A.; Jonsson, C. B.; Surendranathan, S.; Parvathareddy, J.; Blakeley, M. P.; Coates, L.; Louis, J. M.; Bonnesen, P. V.; Kovalevsky, A. Covalent Narlaprevir- and Boceprevir-Derived Hybrid Inhibitors of SARS-CoV-2 Main Protease. *Nat Commun* **2022**, *13* (1), 2268. https://doi.org/10.1038/s41467-022-29915-z.
(31) Mulligan, V. K.; Kang, C. S.; Sawaya, M. R.; Rettie, S.; Li, X.; Antselovich, I.; Craven, T. W.; Watkins, A. M.; Labonte, J. W.; DiMaio, F.; Yeates, T. O.; Baker, D. Computational Design of Mixed Chirality Peptide Macrocycles with Internal Symmetry. *Protein Science* **2020**, *29* (12), 2433–2445. https://doi.org/10.1002/pro.3974.
(32) Fukuzawa, K.; Watanabe, C.; Okiyama, Y.; Nakano, T. How to Perform FMO Calculation in Drug Discovery. *Recent Advances of the Fragment Molecular Orbital Method: Enhanced Performance and Applicability*, 2021, 93–125. https://doi.org/10.1007/978-981-15-9235-5_7.
(33) Watanabe, C.; Watanabe, H.; Okiyama, Y.; Takaya, D.; Fukuzawa, K.; Tanaka, S.; Honma, T. **Development of an Automated Fragment Molecular Orbital (FMO) Calculation Protocol toward Construction of Quantum Mechanical Calculation Database for Large Biomolecules** . *CBIJ* **2019**, *19* (0), 5–18. https://doi.org/10.1273/cbij.19.5.
(34) Takaya, D.; Watanabe, C.; Nagase, S.; Kamisaka, K.; Okiyama, Y.; Moriwaki, H.; Yuki, H.; Sato, T.; Kurita, N.; Yagi, Y.; Takagi, T.; Kawashita, N.; Takaba, K.; Ozawa, T.; Takimoto-Kamimura, M.; Tanaka, S.; Fukuzawa, K.; Honma, T. FMODB: The World's First Database of Quantum Mechanical Calculations for Biomacromolecules Based on the Fragment Molecular Orbital Method. *J. Chem. Inf. Model.* **2021**, acs.jcim.0c01062. https://doi.org/10.1021/acs.jcim.0c01062.
